# A proof of concept for neutralizing antibody-guided vaccine design against SARS-CoV-2

**DOI:** 10.1093/nsr/nwab053

**Published:** 2021-03-27

**Authors:** Li Zhang, Lei Cao, Xing-Su Gao, Bin-Yang Zheng, Yong-Qiang Deng, Jing-Xin Li, Rui Feng, Qian Bian, Xi-Ling Guo, Nan Wang, Hong-Ying Qiu, Lei Wang, Zhen Cui, Qing Ye, Geng Chen, Kui-Kui Lu, Yin Chen, Yu-Tao Chen, Hong-Xing Pan, Jiaping Yu, Wenrong Yao, Bao-Li Zhu, Jianping Chen, Yong Liu, Cheng-Feng Qin, Xiangxi Wang, Feng-Cai Zhu

**Affiliations:** National Health Commission of the People's Republic of China, Key Laboratory of Enteric Pathogenic Microbiology (Jiangsu Provincial Center for Disease Control and Prevention), Nanjing 210009, China; CAS Key Laboratory of Infection and Immunity, National Laboratory of Macromolecules, Institute of Biophysics, Chinese Academy of Sciences, Beijing 100101, China; Center for Global Health, School of Public Health, Nanjing Medical University, Nanjing 211166, China; National Health Commission of the People's Republic of China, Key Laboratory of Enteric Pathogenic Microbiology (Jiangsu Provincial Center for Disease Control and Prevention), Nanjing 210009, China; State Key Laboratory of Pathogen and Biosecurity, Beijing Institute of Microbiology and Epidemiology, Beijing 100071, China; National Health Commission of the People's Republic of China, Key Laboratory of Enteric Pathogenic Microbiology (Jiangsu Provincial Center for Disease Control and Prevention), Nanjing 210009, China; Center for Global Health, School of Public Health, Nanjing Medical University, Nanjing 211166, China; CAS Key Laboratory of Infection and Immunity, National Laboratory of Macromolecules, Institute of Biophysics, Chinese Academy of Sciences, Beijing 100101, China; National Health Commission of the People's Republic of China, Key Laboratory of Enteric Pathogenic Microbiology (Jiangsu Provincial Center for Disease Control and Prevention), Nanjing 210009, China; National Health Commission of the People's Republic of China, Key Laboratory of Enteric Pathogenic Microbiology (Jiangsu Provincial Center for Disease Control and Prevention), Nanjing 210009, China; CAS Key Laboratory of Infection and Immunity, National Laboratory of Macromolecules, Institute of Biophysics, Chinese Academy of Sciences, Beijing 100101, China; State Key Laboratory of Pathogen and Biosecurity, Beijing Institute of Microbiology and Epidemiology, Beijing 100071, China; CAS Key Laboratory of Infection and Immunity, National Laboratory of Macromolecules, Institute of Biophysics, Chinese Academy of Sciences, Beijing 100101, China; CAS Key Laboratory of Infection and Immunity, National Laboratory of Macromolecules, Institute of Biophysics, Chinese Academy of Sciences, Beijing 100101, China; State Key Laboratory of Pathogen and Biosecurity, Beijing Institute of Microbiology and Epidemiology, Beijing 100071, China; National Health Commission of the People's Republic of China, Key Laboratory of Enteric Pathogenic Microbiology (Jiangsu Provincial Center for Disease Control and Prevention), Nanjing 210009, China; National Health Commission of the People's Republic of China, Key Laboratory of Enteric Pathogenic Microbiology (Jiangsu Provincial Center for Disease Control and Prevention), Nanjing 210009, China; National Health Commission of the People's Republic of China, Key Laboratory of Enteric Pathogenic Microbiology (Jiangsu Provincial Center for Disease Control and Prevention), Nanjing 210009, China; CAS Key Laboratory of Infection and Immunity, National Laboratory of Macromolecules, Institute of Biophysics, Chinese Academy of Sciences, Beijing 100101, China; National Health Commission of the People's Republic of China, Key Laboratory of Enteric Pathogenic Microbiology (Jiangsu Provincial Center for Disease Control and Prevention), Nanjing 210009, China; Jiangsu Rec-biotechnology Co. Ltd, Taizhou 225300, China; Jiangsu Rec-biotechnology Co. Ltd, Taizhou 225300, China; National Health Commission of the People's Republic of China, Key Laboratory of Enteric Pathogenic Microbiology (Jiangsu Provincial Center for Disease Control and Prevention), Nanjing 210009, China; Jiangsu Rec-biotechnology Co. Ltd, Taizhou 225300, China; Jiangsu Rec-biotechnology Co. Ltd, Taizhou 225300, China; State Key Laboratory of Pathogen and Biosecurity, Beijing Institute of Microbiology and Epidemiology, Beijing 100071, China; CAS Key Laboratory of Infection and Immunity, National Laboratory of Macromolecules, Institute of Biophysics, Chinese Academy of Sciences, Beijing 100101, China; Guangzhou Regenerative Medicine and Health Guangdong Laboratory, Guangzhou 510200, China; National Health Commission of the People's Republic of China, Key Laboratory of Enteric Pathogenic Microbiology (Jiangsu Provincial Center for Disease Control and Prevention), Nanjing 210009, China; Center for Global Health, School of Public Health, Nanjing Medical University, Nanjing 211166, China

**Keywords:** SARS-CoV-2, neutralizing antibodies, vaccine development, structure-based immunogen design

## Abstract

Mutations and transient conformational movements of the receptor binding domain (RBD) that make neutralizing epitopes momentarily unavailable present immune escape routes for severe acute respiratory syndrome coronavirus 2 (SARS-CoV-2). To mitigate viral escape, we developed a cocktail of neutralizing antibodies (NAbs) targeting epitopes located on different domains of spike (S) protein. Screening of a library of monoclonal antibodies generated from peripheral blood mononuclear cells of COVID-19 convalescent patients yielded potent NAbs, targeting the N-terminal domain (NTD) and RBD domain of S, effective at *nM* concentrations. Remarkably, a combination of RBD-targeting NAbs and NTD-binding NAbs, FC05, enhanced the neutralization potency in cell-based assays and an animal model. Results of competitive surface plasmon resonance assays and cryo-electron microscopy (cryo-EM) structures of antigen-binding fragments bound to S unveil determinants of immunogenicity. Combinations of immunogens, identified in the NTD and RBD of S, when immunized in rabbits and macaques, elicited potent protective immune responses against SARS-CoV-2. More importantly, two immunizations of this combination of NTD and RBD immunogens provided complete protection in macaques against a SARS-CoV-2 challenge, without observable antibody-dependent enhancement of infection. These results provide a proof of concept for neutralization-based immunogen design targeting SARS-CoV-2 NTD and RBD.

## Teaser

Immunogens identified in the N-terminal domain and receptor binding domain of the severe acute respiratory syndrome coronavirus 2 (SARS-CoV-2) spike protein using a cocktail of non-competing neutralizing antibodies when injected in macaques elicited a potent protective efficacy against SARS-CoV-2.

## INTRODUCTION

Coronavirus disease 2019 (COVID-19), caused by the severe acute respiratory syndrome coronavirus 2 (SARS-CoV-2), continues to spread across the world since December 2019 [1,2]. The 27 January 2021 World Health Organization (WHO) Situation Report cited over 100 million COVID-19 cases and 2 164 627 deaths. These numbers continue to rise daily [3]. Concerningly, various variants of SARS-CoV-2 carrying D614G or N501Y or other spike mutations, which seemingly enhance infectivity, have been documented and are fast becoming the dominant strains of SARS-CoV-2 globally [4]. Safe and effective preventive as well as therapeutic measures are urgently needed to bring the ongoing pandemic of COVID-19 under control [5]. Over the past several months, experimental and clinical strategies based on eliciting neutralizing antibodies (NAbs) via immunization of potential vaccine candidates and passive administration of NAbs have shown promise in protecting and curing SARS-CoV-2 infections [6–8]. The successes of these studies highlight the importance of screening and identification of immunogens capable of eliciting high NAb titers. Furthermore, NAbs elicited by immunogens differ significantly in their abilities to neutralize SARS-CoV-2 and confer protection. Therefore, a deep understanding of the nature of NAbs capable of potently neutralizing SARS-CoV-2 and their epitopes could guide new approaches for the development of vaccines.

The coronavirus spike (S) protein is a multifunctional molecular machine that facilitates viral entry into target cells by engaging with cellular receptors, and determines to a great extent cell tropism and host range [9]. Coronaviruses S proteins are processed into S1 and S2 subunits by host proteases, among which S1 is responsible for receptor binding, while the S2 subunit mediates membrane fusion [10]. The S1 subunit typically possesses two types of domains capable of binding to host cell receptors. For instance, some betacoronaviruses use the N-terminal domain (NTD) of their S1 subunit to bind sialic acids located on the glycosylated cell-surface receptor [11]. Similarly, the betacoronavirus murine hepatitis virus uses its NTD for binding the protein receptor CEACAM1 [12]. In contrast to this, SARS-CoV and SARS-CoV-2 use the C-terminal domain (named also as receptor binding domain, RBD) of their S1 subunit for binding to their protein receptor hACE2 [13]. Recently, the NTD of SARS-CoV-2 has also been shown to be involved in ACE2-dependent entry into host cells by targeting the high-density lipoprotein (HDL) scavenger receptor B type 1 (SR-B1) [14]. In line with this, a number of antibodies targeting the NTD exhibit potent neutralizing activities against SARS-CoV-2 and Middle East respiratory syndrome coronavirus (MERS-Cov) infections [15,16]. Abrogation of the crucial role played by the S in the establishment of an infection is the main goal of therapies based on neutralizing antibodies and the focus of antibody-based drug and vaccine design. More recently, a number of RBD-targeting NAbs against SARS-CoV-2, which block the binding of the S trimer to hACE2 and/or viral membrane fusion as well as possessing other neutralizing mechanisms, have been reported and characterized [17–31]. Stochastic conformational movements of the RBD transiently expose or hide the determinants of receptor binding and some key neutralizing epitopes, which might open up fortuitous escape routes for the virus. Furthermore, antibody-mediated selective pressure is known to lead to antigenic drift within the RBD, resulting in the accumulation of mutations that hamper neutralization by antibodies [17,18]. To address these issues related to SARS-CoV-2 neutralization, administration of a cocktail of NAbs targeting both the RBD and non-RBD regions, rather than using a single NAb, could potentially increase the potency of protection via binding of NAbs to multiple domains of S, thereby preventing escape of viral particles from the NAbs. In this context, the immunogenic characteristics of the antigens targeted by potential NAb cocktails and their structural features can inform strategies for the development of vaccines and therapeutics against COVID-19.

## RESULTS

**Figure 1.**
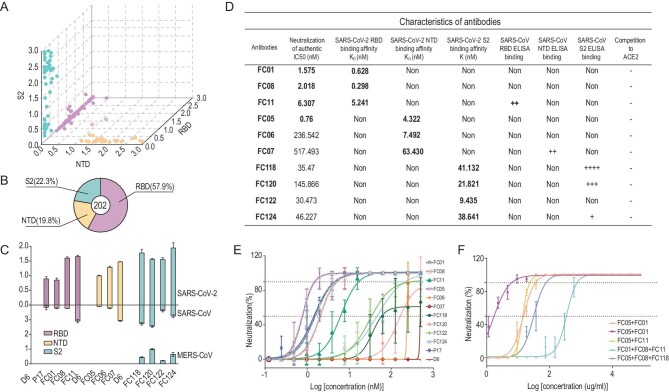
Identification and characterization of SARS-CoV-2 S-targeting neutralizing antibodies. (A) Characteristics of antibodies binding to SARS-CoV-2 RBD, NTD or S2 by ELISA. SARS-CoV-2 RBD-, NTD- and S2-specific mAbs are colored in pink, wheat and pale cyan dots, respectively. A number of mAbs that exhibit non-specific bindings to both SARS-CoV-2 RBD and NTD are presented in wheat dots with pink outlines. (B) Proportion of SARS-CoV-2 S-specific antibodies targeting each of the indicated domains. (C) Bar graph depicting the binding of 10 representative mAbs (FC01, FC05, FC06, FC07, FC08, FC11, FC118, FC120, FC122 and FC124; positive control antibody-P17 and negative control antibody-D6 were used in the assay) to S proteins of SARS-CoV-2, SARS-CoV and MERS-CoV using ELISA assays (shown as mean ± SD of values derived from experiments conducted in triplicate). (D) Summary of the performance of the representative 10 mAbs in the indicated assays. All listed antibodies in Fig. 1D were tested for their ability to compete with hACE2 and the ‘-’ means ‘no competition’. *In vitro* neutralization activities of (E) 10 individual mAbs or (F) the cocktail of antibodies against SARS-CoV-2 in Vero-E6 cells. Positive (P17) [22] and negative (D6, EV71 antibody) [38] controls were used in the neutralization assay. Neutralizing activities are represented as mean ± SD. Experiments were performed in triplicates. The lower dotted lines indicate the IC_50_ values, and the upper ones indicate the IC_90_ values.

One of the prospective goals of this study was to generate a large and diverse collection of human NAbs targeting multiple domains of S so as to allow for the formulation of a cocktail of highly potent antibodies that could simultaneously bind to the various regions of S. For this, we first established antigen-binding fragment (Fab) phage-display libraries from the peripheral blood mononuclear cells (PBMCs) of five COVID-19 convalescent patients. After three rounds of panning, ∼350 randomly picked colonies were screened by enzyme-linked immunosorbent assay (ELISA) for binding to the SARS-CoV-2 S trimer. A set of 202 positive Fab clones exhibiting tight binding to SARS-CoV-2 were selected for sequencing and further analysis (Fig. 1A). We evaluated the ability of these Fabs to bind to recombinant SARS-CoV-2 NTD, RBD or S2 proteins and observed that 40 (∼20%), 117 (58%) and 45 (22%) of these monoclonal antibodies (mAbs) recognized the NTD, RBD and S2, respectively (Fig. 1A and B). We then narrowed down our selection of candidates for the development of a cocktail to 10 antibodies picked from each of these three groups based on their binding affinities and genetic diversity assessed from the phylogenetic analysis performed using the amino acid sequences of the VH-D-JH and VL-JL regions [19] (Fig. 1A, Figs S1 and S2, and Table S1). Among those selected, three (named FC01, FC08 and FC11) target the RBD, three (named FC05, FC06 and FC07) recognize the NTD and four (named FC118, FC120, FC122 and FC124) are S2-specific mAbs (Fig. 1C and D). To find out whether these antibodies cross-react with SARS-CoV and MERS-CoV, we firstly assessed the binding capacity of these mAbs to RBDs, NTDs and S2s from SARS-CoV-2, SARS-CoV and MERS-CoV by ELISA (Fig. 1C). FC11 and FC07 showed cross-binding to the SARS-CoV RBD and NTD, respectively. Expectedly, the four S2-directed mAbs interacted with S2s from all three viruses, of which FC122 bound weakly to SARS-CoV and MERS-CoV (Fig. 1C). Surface plasmon resonance (SPR) assays demonstrated that all 10 mAbs exhibit tight bindings to SARS-CoV-2 with affinities in the range of 0.3–62 nM (Fig. 1D). Interestingly, binding affinities of S2-targeting mAbs were relatively weaker than those of RBD- or NTD-targeting antibodies (Fig. 1D and Fig. S3). Sequence alignments of these representative mAbs with currently reported NAbs against SARS-CoV-2 reveal hyper diversities in light chain complementarity-determining region (CDR) 1 and heavy chain CDR3 (Fig. S4).

The effectiveness of the neutralization abilities of the 10 mAbs against SARS-CoV-2 infection when tested using Vero-E6 cells revealed that all 10 showed neutralizing activities with IC_50_ values ranging from 0.8–520 nM, among which the three RBD-targeting and one NTD-binding (FC05) mAbs potently neutralized the virus at nM levels (Fig. 1E). These results, together with the results of the binding site studies, allowed us to rationally evaluate the neutralization potency of the NTD-targeting FC05 in combination with the RBD-targeting NAbs. Not surprisingly, the combination of any one of the RBD-targeting NAbs and FC05 enhanced the neutralization potency dramatically when compared to neutralization performed by using individual NAbs under identical conditions (Fig. 1F). Notably, the cocktail consisting of FC05 (NTD-binding) and FC08 (RBD-binding) yielded the strongest neutralizing activity with an IC_50_ value as low as 15 pM, which was better than the cocktail consisting of FC05 and FC01 as well as other combinations of three or four NAbs (Fig. 1F). Although more recently, synergistic effects between pairs of non-competing RBD-targeting NAbs have been reported for SARS-CoV-2 [17,20–22], our cocktail of FC05 and FC08 that bind to different domains of the S trimer provides a proof of concept for neutralization-based immunogen design targeting both SARS-CoV-2 NTDs and RBDs.

**Figure 2.**
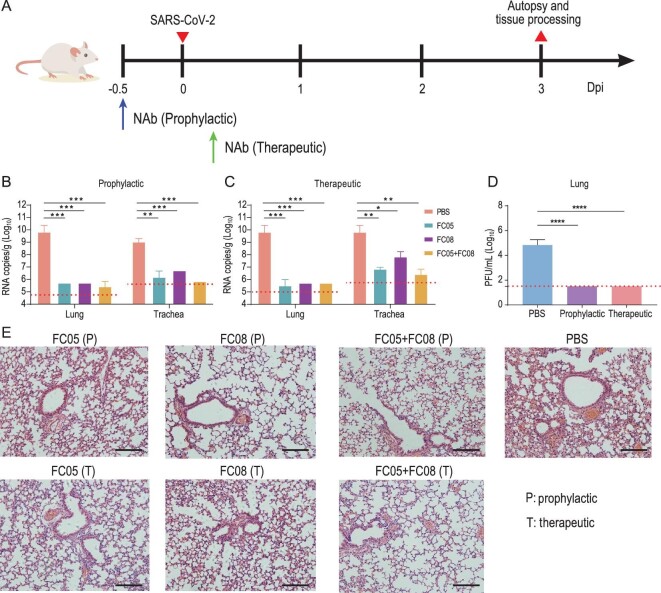
Prophylactic and therapeutic efficacy of FC05, FC08 or the cocktail of antibodies of FC05 and FC08 in SARS-CoV-2 susceptible mouse model. (A) Experimental design for therapeutic and prophylactic evaluations of FC05, FC08 or the cocktail of antibodies consisting of FC05 and FC08 by using a mouse-adapted SARS-CoV-2 virus (MASCp6) in mouse model. Group of BALB/c mice were infected intranasally with 2 × 10^4^ plaque forming unit (PFU) of MASCp6. A dose of 20 mg/kg of antibody was administrated intraperitoneally 12 h before infection (the prophylactic group, P) or at 2 h after infection (the therapeutic group, T). Phosphate buffer saline (PBS) injection was used as a negative control group. Then, the lung and the trachea tissues of mice were collected at 3 and 5 dpi for virus load measurement and histopathological analysis. (B) and (C) Virus loads of lung and trachea tissues at 3 dpi in mouse model. The viral loads of the tissues were determined by quantitative reverse transcription polymerase chain reaction (qRT-PCR) (****P < 0.0001). Data are represented as mean ± SD. Dashed lines represent the limit of detection. (D) Virus loads of the lung. RNA was extracted and viral load was determined by a plaque assay of lung tissue homogenates (*P < 0.05; **P < 0.01; ***P < 0.001). Data are represented as mean ± SD. Dashed lines represent the limit of detection. (E) Histopathological analysis of lung and trachea samples at 3 dpi. Scale bar: 100 μm.

Next, we sought to assess the *in vivo* protection efficacy of these NAbs against a SARS-CoV-2 challenge. A newly established mouse model based on the SARS-CoV-2 mouse-adapted strain MASCp6 [23] was used to evaluate the potential prophylactic and therapeutic efficacy of these NAbs. Bagg's albino/c (BALB/c) mice were administered a single dose of 20 mg/kg of FC05 or FC08 or a cocktail of FC05 (NTD-binding) and FC08 (RBD-binding) either 12 h before (day −0.5) or 0.5 day (day 0.5) after viral challenge with 2 × 10^4^ PFU of MASCp6 (BetaCoV/Beijing/IMEBJ05-P6/2020) (Fig. 2A). Animals were sacrificed at day 3 for detecting viral loads and examining the pathology of the lungs and tracheas. The number of viral RNA copies estimated in the lungs and tracheas revealed that, in prophylactic settings, a treatment with either individual NAbs or the cocktail led to a 3–4 log reduction of viral loads in both lungs and tracheas at day 3 when compared to the PBS-treated group. A modest synergistic protective efficacy was observed for the cocktail (Fig. 2B and C). The estimated viral loads from the lungs of groups belonging to therapeutic settings showed similar levels to those observed for the groups of the prophylactic settings, however, the viral loads from the tracheas differed for both the groups. A ∼10-fold higher titer was observed for the groups in therapeutic settings (Fig. 2B and C). Notably, all mice from FC05/FC08/FC05 and FC08-treated groups no longer had infectious virus in the lungs at day 3 as measured by a plaque assay of lung tissue homogenates (Fig. 2D). More importantly, histopathological examination revealed a typical interstitial pneumonia, including widening of alveolar septum, vasodilation, hyperemia and edema, accompanied by a large number of monocytes and lymphocytes and a small number of lobulated granulocytes and other inflammatory cell infiltration in mice belonging to the PBS control group (Fig. 2E). In contrast, no obvious lesions of alveolar epithelial cells or focal hemorrhage were observed in the lung sections of either of the antibody-treated groups at day 3 (Fig. 2E). Although numerous antibody cocktails with various synergistic neutralization potencies exhibited at cellular levels have been reported [22,24,25], none of these cocktails have been reported to exhibit substantial synergistic protection in animals, indicative of a challenge to observe genuine synergy. This is more likely due to the lack of ideal animal models.

**Figure 3.**
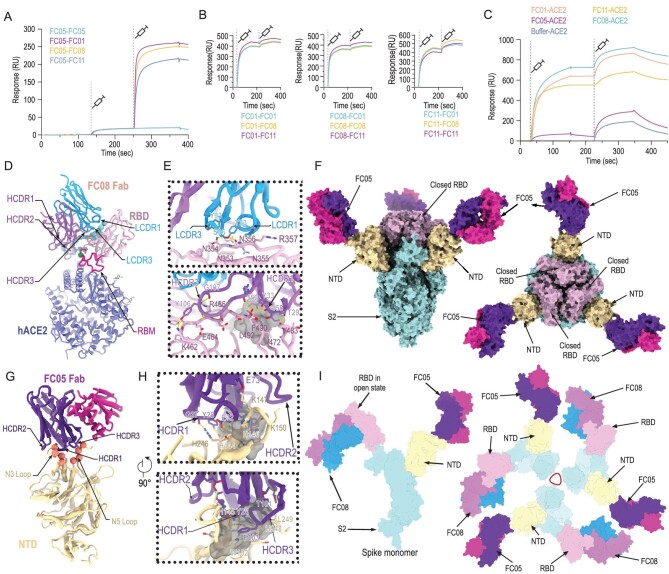
Epitope mapping of SARS-CoV-2 NAbs. (A) SPR kinetics of simultaneous binding of FC05 and three RBD-directed NAbs to SARS-CoV-2 S trimer. (B) SPR-based competitive binding of three RBD-directed NAbs to SARS-CoV-2 S trimer. SARS-CoV-2 S trimer was initially immobilized onto the sensor. One NAb was first injected, followed by the other two, which indicated that these three RBD-directed NAbs competed with each other for binding to the SARS-CoV-2 S trimer. (C) Binding of any one RBD-directed NAb blocks the interactions between ACE2 and SARS-CoV-2 S trimer as assessed by competitive SPR. (D) Structure of SARS-CoV-2 S RBD-FC08-hACE2 complex. The light chain of FC08 Fab is shown in cyan, and the heavy chain in magenta. The RBD and hACE2 are colored in pink and blue, respectively. Residues that constitute the FC08 epitope and the RBM are shown as spheres and colored in green and blue, respectively. The RBM is shown in red. (E) Details of the interactions between the FC08 and SARS-CoV-2 RBD. Some residues involved in the formation of hydrophobic patches (gray mesh) and hydrogen bonds (yellow dash) are shown as sticks and labeled. (F) Cryo-EM structure of SARS-CoV-2 S trimer-FC05 complex. Each S monomer is depicted by various colors and the FC05 Fabs are shown in hot pink (light chains) and purple-blue (heavy chains). (G) Interactions between the NTD and FC05. The loops involved in interactions with FC05 are labeled. (H) Details of the interactions between the FC05 and SARS-CoV-2 NTD. (I) The proposed model of SARS-CoV-2 S trimer in complex with FC05 and FC08. Color scheme is the same as in (D) and (F).

To gain a better understanding of the synergy observed during the neutralization of SARS-CoV-2 by a cocktail of NAbs, we performed competitive SPR assays. The results of the assays were expected to reveal whether the NAbs recognize the same or different patches of the epitopes. As expected, the binding of the NTD-specific FC05 does not affect the attachment of any of the three RBD-specific NAbs to the SARS-CoV-2 S trimer, explaining the cooperativity between the antibodies of the cocktail as they bind simultaneously to distinct domains (Fig. 3A). Conversely, the three RBD-targeting NAbs competed with each other for binding to the SARS-CoV-2 S trimer (Fig. 3B), which may imply that these RBD-targeting antibodies recognize similar epitopes or their epitopes overlap partially. Lastly, none of the above four NAbs were capable of blocking the interactions between soluble hACE2 and the SARS-CoV-2 S trimer, suggesting a distinct neutralizing mechanism (Fig. 3C). To decipher the nature of the epitopes and the mechanism of neutralization at the atomic level, we determined cryo-EM structures of a prefusion stabilized SARS-CoV-2 S ectodomain trimer in complex with the Fab fragments of the NAbs. Surprisingly, the structural studies revealed that all three RBD-targeting NAbs were capable of breaking the S trimer into monomers or irregular pieces. A similar perturbation of the S trimer was observed previously in the studies conducted on the CR3022 antibody [26]. The NTD-binding FC05, however, neither exhibited any such ability to disrupt the S trimer nor affected the viral stability (Fig. S5). To further map epitope clusters of these RBD-binding NAbs, we used a representative antibody, FC08, for performing a competitive SPR-based epitope binding assay. Recently, we mapped the antigenic sites of three well-characterized RBD-targeting NAbs, H014, HB27 and P17 [22,27,28], which bind epitopes located on one side of the RBD, the apical head of the RBD and the receptor binding motif (RBM), respectively. Using these previously characterized antibodies along with FC08 in the assays revealed that only P17 competes with FC08 for binding to the SARS-CoV-2 S trimer (Fig. S6). Taking into consideration the observation that FC08 does not seem to target the RBM due to the simultaneous bindings of FC08 and the hACE2 to SARS-CoV-2 S trimer (Fig. S6), FC08 probably targets a cryptic epitope that partially overlaps with P17 and probably lies towards the interior of the S trimer.

To gain an understanding of the molecular basis of the destruction of S trimer by FC08, structural investigations of the SARS-CoV-2 RBD in complex with FC08 were carried out. A non-competing Fab and hACE2 that recognize the RBD beyond FC08 binding sites were used to increase the molecular weight of this complex for pursuing a high resolution (up to 3.6 Å) structure by cryo-EM reconstruction (Figs S7 and S8, and Table S2). As revealed by competitive SPR analysis, FC08 recognizes a cryptic epitope on the other side of the RBD, adjacent to the hACE2-binding site (Fig. 3D). The FC08 epitope is inaccessible in the prefusion S trimer (neither the open nor the closed RBDs), suggesting that FC08 binding would facilitate conversion to the disassembly states (Fig. S9), which explains its neutralization of SARS-CoV-2 via destruction of the prefusion S trimer. FC08 uses both heavy and light chains, and five of six CDR loops for interaction with the RBD (Fig. 3E). The buried surface area on the epitope is 1057 Å^2^ and SARS-CoV-2 recognition by FC08 is largely driven by hydrophobic interactions (Fig. 3E). The FC08 epitope consists of 24 residues, primarily located in the β1 (residues 353–357), β5 (residues 449 and 452), β6 (residues 490 and 492) and the loop between β5 and β6 (residues 462–472 and 481–484) (Fig. S10 and Table S3). Among these, 11 residues (46%) are not conserved between SARS-CoV-2 and SARS-CoV, explaining its specificity for SARS-CoV-2 for binding and neutralization (Fig. S10). Notably, the residue 501, identified as a key mutation site in recently isolated SARS-CoV-2 variants (such as N501Y) and shown to substantially enhance interaction with hACE2, is not involved in the interactions with FC08. These results indicate that FC08 has the potential to neutralize the recent more-contagious SARS-CoV-2 variants.

Cryo-EM characterization of the S-FC05 complex showed full occupancy where one Fab was bound to each NTD of the homotrimeric S (Fig. 3F). 3D classification revealed that the S trimer adopts a 3-fold symmetrical structure with all three RBDs closed, albeit without imposing any symmetry. By applying a C3 symmetry, we reconstructed the cryo-EM structure of the complex at an overall resolution of 3.4 Å. However, the electron potential map for binding interface between NTD and FC05 is relatively weak due to conformational heterogeneity. To solve this problem, focusing classification and refinement by using a ‘block-based’ reconstruction approach [29] was used to improve the local resolution to 3.9 Å (Fig. 3F, Figs S11–S13 and Table S2). Interestingly, the binding mode of FC05 resembles that of 4A8, a recently reported SARS-CoV-2 NAb [15] (Fig. S14). Similar to 4A8, FC05 recognizes a conformational epitope formed by elements of the N3 and N5 loops located on the NTD with a buried surface area of ∼700 Å^2^. The essential epitope contains 12 residues, among which all 12 residues (100%) are not conserved between SARS-CoV and SARS-CoV-2, explaining FC05’s virus-specific binding and neutralization activities (Fig. 3G and H, and Fig. S15). The paratope of FC05 is composed of four CDR loops: CDRL2 (residues 49–55), CDRH1 (residues 29–33), CDRH2 (residues 50–59) and CDRH3 (residues 99–106) (Table S4). Extensive hydrophobic and hydrophilic interactions facilitate the tight binding between FC05 and the NTD.

Despite the failure to reconstruct the structure of the complex of the cocktail of antibodies containing NTD-binding FC05 and RBD-binding FC08 with the S trimer, we tried to answer the question of whether the NTD-binding FC05 could function as a potential partner of RBD-targeting NAbs for formulating a cocktail, by modeling the structure of SARS-CoV-2 S trimer in complex with FC05 and FC08. Each S monomer harbors distinct binding sites for FC05 and FC08, separately. There are in total six copies of the cocktail of NAb Fabs bound to one S trimer, where three FC05 and three FC08 Fabs bind on the side of each NTD and each RBD, respectively, shielding most of the regions of S1 as well as dispersing the S trimer (Fig. 3I). These structural studies provide clues for unraveling the molecular basis of the synergic neutralization of SARS-CoV-2 by the cocktail of antibodies that are capable of simultaneously targeting both NTDs and RBDs. In addition, this combination of FC05 and FC08 could be complemented in synergy by an antibody that blocks hACE2 attachment, such as HB27, further reinforcing the cocktail as a SARS-CoV-2 therapeutic.

Most of the potent neutralizing antibodies reported to date target the RBD of CoV [20,27,30]. Therefore, a number of RBD-subunit-based vaccines for protection against SARS, MERS and COVID-19 are under development [31–34]. However, RBD-subunit-based vaccines could face some critical challenges arising from their relatively low immunogenicity, low diversity within the elicited antibodies and the ensuing potential escape of viral mutants from the antibodies under selective pressure. Our study here, together with other recently published studies, indicates that a subset of NTD-directed antibodies possesses potent neutralizing activities (Figs 1 and 2) and that cocktails of antibodies containing NTD-directed as well as RBD-targeting NAbs act in synergy to confer protection against SARS-CoV-2, suggesting that the NTD is a promising immunogenic partner of the SARS-CoV-2 RBD. In addition, vaccination with NTD protein could elicit NAbs and NTD-specific T cell responses, protecting lungs from pathology in a MERS-CoV challenge animal model [35]. The integration of the NTD in an RBD-based COVID-19 vaccine would increase NAb diversity and decrease the potential of viral escape of host immunity. To verify this idea, SARS-CoV-2 NTD (residues 1–305) and RBD (residues 319–541) were expressed in mammalian cells, purified by size exclusion chromatography, and their antigenicity was characterized by using the NAbs identified in this study. ELISA assays revealed these NAbs possessed comparable binding activities to the recombinant NTD or RBD when compared to the S trimer (Fig. S16).

**Figure 4.**
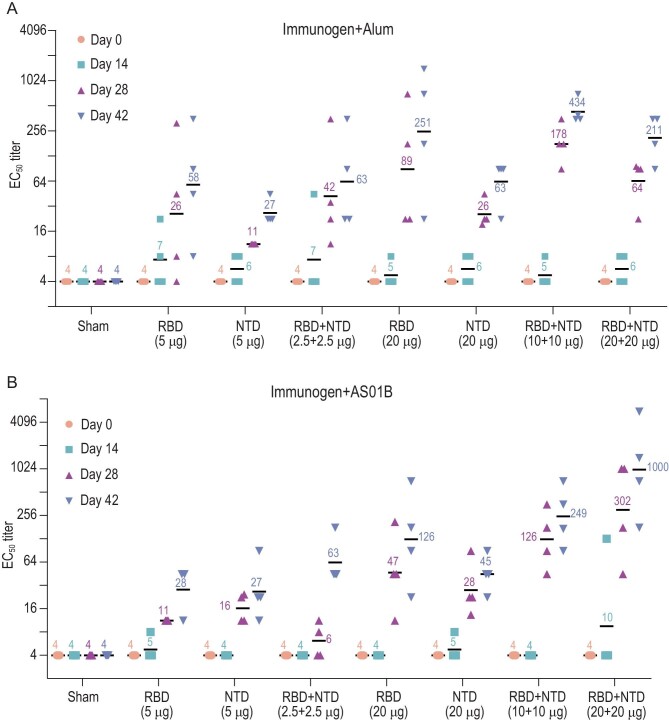
Immunogenic evaluation of candidate antigen formulations mixed with alum or AS01B adjuvant in rabbits. (A and B) Rabbits were immunized intramuscularly with various doses of individual (RBD or NTD) or combined (RBD + NTD) immunogens mixed with alum or AS01B adjuvant or adjuvant only (sham) (*n* = 4). Neutralizing antibody titer against live SARS-CoV-2 was measured. Data points represent mean ± SEM of individual rabbits from four independent experiments; error bars reflect SEM; horizontal lines indicate the geometric mean titer (GMT) of EC_50_ for each group.

To evaluate the potential of the recombinant NTD and RBD as vaccine candidates, 16 groups of New Zealand rabbits (*n* = 4/group) were injected at day 0, 14 and 28 with various doses of candidate antigen formulations mixed with alum or AS01B adjuvant as follows: 5 μg RBD or 5 μg NTD or 2.5 μg RBD + 2.5 μg NTD; or 20 μg RBD or 20 μg RBD or 10 μg RBD + 10 μg NTD; or 20 μg RBD + 20 μg NTD per dose, 0 μg of antigens in physiological saline as the sham group (Fig. 4). No inflammation or other adverse effects were observed in the animals. Titers of SARS-CoV-2-specific neutralizing antibodies produced by the animals over a period of time (at week 0, 2, 4 and 6), effective in neutralizing the live virus, were monitored using microneutralization assays (MN50). Similar to the immune responses elicited by an inactivated SARS-CoV-2 vaccine candidate (PiCoVacc) reported by us previously, the neutralizing antibody titer emerged at week 2, surged at week 4 and continued to increase at week 6 (Fig. 4). Perhaps correlated with the lower glycosylation and more neutralizing epitopes present in the RBD, the RBD induced much higher NAb titers than the NTD at various doses. However, the combined immunogens of the RBD and NTD (ReCovR+N) exhibited more robust and stable immunogenicity for neutralization compared with a single immunogen consisting of either the RBD or NTD at the same dose tested under identical conditions (Fig. 4). Notably, relatively large differences in the ability of individual animals in eliciting NAb titers were observed within the RBD vaccinated groups. The addition of the NTD to RBD not only substantially enhanced the NAb titer, but also remarkably decreased the fluctuation in eliciting NAb titer from immunized animals (Fig. 4). Compared to AS01B, alum-based adjuvant facilitates the antigens in boosting immune responses at 5 or 20 μg/dose. Administration of higher doses of ReCovR+N (20 μg RBD + 20 μg NTD) with AS01B led to the highest NAb titer, up to ∼1000. In contrast, immunization with higher doses of ReCovR+N in conjunction with alum yielded a decreased NAb titer (∼200) compared to the median dose (10 μg RBD + 10 μg NTD), indicative of the need for proper collocation of the adjuvant and various doses of antigen during immunization (Fig. 4).

**Figure 5.**
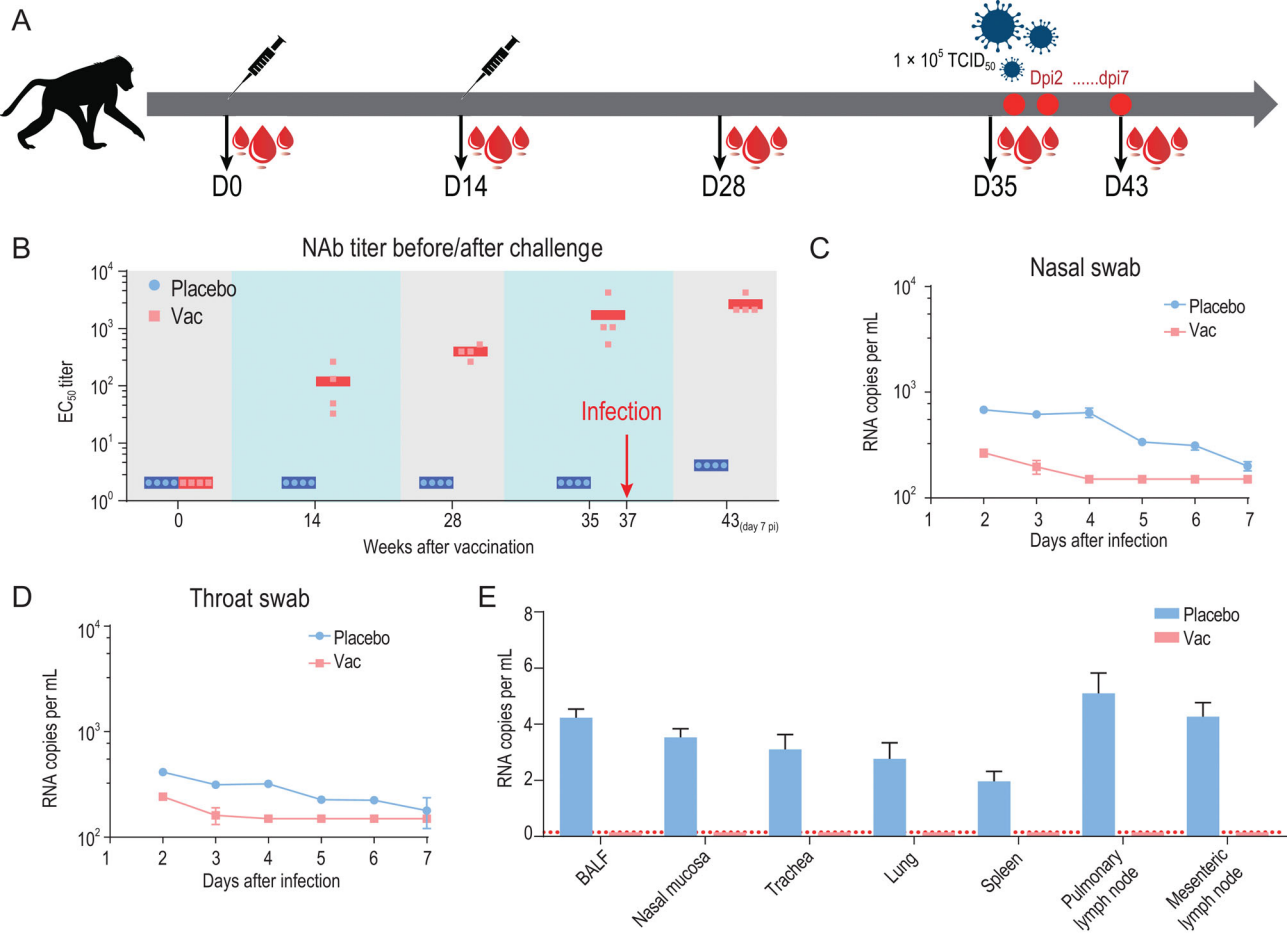
Immunogenicity and protective efficacy of ReCovR+N in rhesus macaques. (A) Schematic diagram of ReCovR+N immunization and sample collection. (B) SARS-CoV-2-specific NAb titer was measured. Data points represent mean ± SD of individual macaques from four independent experiments. Error bars indicate SEM. Horizontal lines indicate the geometric mean titer of EC_50_ for each group. Viral loads of (C) nasal and (D) throat swab specimens collected from the inoculated macaques at 2, 3, 5, 6 and 7 dpi were monitored. (Ε) Viral loads in various tissues from all the challenged macaques at 7 dpi were measured. RNA was extracted and viral load was determined by qRT-PCR. All data are presented as means ± SD from four independent experiments. Dotted lines indicate the limit of detection.

We next evaluated the immunogenicity and protective efficacy of ReCovR+N in rhesus macaques. Macaques were immunized two times via the intramuscular route with ReCovR+N (20 μg RBD + 20 μg NTD) mixed by AS01B adjuvant or placebo (PBS + AS01B) at day 0 and 14 (*n* = 4) (Fig. 5A). SARS-CoV-2 specific NAbs emerged at week 2 and rose up to ∼2000 at week 5 (before virus challenge) in the vaccinated group, whose titer was ∼20-fold higher than that of serum from the PiCoVacc immunized group (Fig. 5B). Subsequently, a challenge study by a direct infection of 10^5^ TCID_50_ of SARS-CoV-2 through intranasal route at day 35 (one week after the second immunization) in vaccinated and control macaques was conducted to verify the protective efficacy. As expected, all control macaques exhibited massive copies of viral genomic RNA in nasal and throat swabs as well as many organs, such as lungs by day 3–7 post-infection (dpi) (Fig. 5C–E). In contrast, viral loads decreased significantly during early infection and were not detected any more by 4–7 dpi in all vaccinated macaques (Fig. 5C–E). In addition, no antibody-dependent enhancement of infection (ADE) was observed for any vaccinated macaques. Collectively, ReCovR+N can confer effective protection in non-human primates against SARS-CoV-2 strains by eliciting potent humoral responses.

COVID-19 vaccine development is moving at an unprecedented speed, with more than 250 candidates under development worldwide. S protein constitutes the main immunogen of a COVID-19 vaccine. While S protein is relatively metastable when produced as a recombinant protein, it is prone to conversion from the prefusion to the postfusion state, losing its immunogenicity [36]. The S protein can be cleaved into S1 and S2 subunits, the former consisting of the NTD, RBD and two subdomains (SD1 and SD2) [28]. Interestingly, most potent NAbs target either the RBD or the NTD, rather than other domains, indicating that the NTD and RBD might be *bona fide* immunogens for eliciting neutralizing antibodies. Selection of the target immunogen is critical for the success of a vaccine, since eliciting a large amount of antibody that binds, but does not neutralize, may lead to low protection or even immunopathology [37]. The ideal immunogen should elicit high-quality, functionally neutralizing antibodies while avoiding induction of non-neutralizing antibodies. The strategy for immunization with a combination of the NTD and RBD, to our knowledge, entails two conceptual advantages: (i) epitope focusing to elicit high levels of NAbs; (ii) optimal/flexible ratio between the NTD and RBD for immunization, rather than the 1 : 1 ratio observed in the S1 subunit, for preferentially inducing higher titers of desirable NAbs, according to the various immunogenic characterizations of the NTD and RBD. We are now entering a new era of precision vaccinology in which multi-disciplinary techniques provide avenues to rapidly isolate and characterize human NAbs, to define the structural basis of antigenicity, to understand mechanisms of viral neutralization and to guide rational immunogen design. The immunogenic data derived from immunization with a combination of SARS-CoV-2 RBD and NTD here demonstrate the feasibility of eliciting robust targeted immune profiles by using antibody-guided vaccine design, and advance us a step forward towards a future of precision vaccines.

## Data and materials availability

Cryo-EM density maps have been deposited at the Electron Microscopy Data Bank with accession codes EMD-30573 (SARS-CoV-2 S-FC05) and EMD-30895 (RBD-FC08-D14-hACE2 complex), and related atomic models have been deposited in the protein data bank under accession code 7D4G and 7DX4, respectively.

## Supplementary Material

nwab053_Supplemental_File
