# Supplementary material for: A proof of concept for neutralizing antibody-guided vaccine design against SARS-CoV-2
Source: Natl Sci Rev. 2021 Mar 27;8(8):nwab053. doi: 10.1093/nsr/nwab053 (PMC8083607; doi:10.1093/nsr/nwab053)
Supplement: nwab053_Supplemental_File [file nwab053_supplemental_file.docx]

Supplementary Materials for

**A proof of concept for neutralizing antibody-guided vaccine design against SARS-CoV-2**

Li Zhang^1$^, Lei Cao^2$^, Xing-Su Gao^4$^, Bin-Yang Zheng^1$^, Yong-Qiang Deng^3$^, Jing-Xin Li^1,4$^, Rui Feng^2^, Qian Bian^1^, Xi-Ling Guo^1^, Nan Wang^2^, Hong-Ying Qiu^3^, Lei Wang^2^, Zhen Cui^2^, Qing Ye^3^, Geng Chen^1^, Kui-Kui Lu^1^, Yin Chen^1^, Yu-Tao Chen^2^, Hong-Xing Pan^1^, Jiaping Yu^6^, Wenrong Yao^6^, Bao-Li Zhu^1^, Jianping Chen^6^, Yong Liu^6^, Cheng-Feng Qin^3^*, Xiangxi Wang^2,5^*, Feng-Cai Zhu^1,4^*

*Correspondence to: X.W. (Email: xiangxi@ibp.ac.cn) or C.F.Q. (Email: qincf@bmi.ac.cn) or F.Z. (Email: jszfc@vip.sina.com)

Materials and Methods

Figs. S1 to S16

Tables. S1 to S3

Supplementary References and Notes

**Materials and Methods**

**Ethics statement**

The informed consent from 5 donors was obtained for the use of blood and blood components. The study was commenced after the approval from the Institutional Review Board of the Jiangsu Provincial Center of Disease Control and Prevention. The protocol and procedures used in the studies with rabbits were reviewed and approved by the Laboratory Animal Welfare and Ethics Committee in the Jiangsu Provincial Center of Disease Control and Prevention. All animal procedures were reviewed and approved by the Animal Experiment Committee of Laboratory Animal Center, Academy of Military Medical Sciences (AMMS), China (Assurance Number: IACUC-DWZX-2020-001).

**Cells**

Vero-E6 cells were cultured in Dulbecco’s Modified Eagle Medium (DMEM, Gibco, 12430-054) containing 10% fetal bovine serum (FBS, Gibco, 12657-029), 100 units/mL of Penicillin and 0.1 mg/mL of Streptomycin (Invitrogen) (Gibco, 15140-122) at 37℃ under 5% CO_2_. HEK-293F cells were suspension-adapted and maintained in FreeStyleTM 293 Expression Medium (Gibco, 12338-018) supplemented with 100 units/ml of Penicillin and 0.1 mg/ml of Streptomycin (Invitrogen) (Gibco, 15140-122) at 37°C in an orbital incubator shaker with 8% CO_2_. Vero-E6 cells were used for the amplification and estimation of the titer of the live virus by JSCDC. HEK-293F cells were employed for the expression of RBD and NTD antigens by the Institute of Biophysics of Chinese Academy of Sciences. HEK-293F cells were used for the expression of the RBD, NTD and S2 antibodies at JSCDC.

**Virus generation**

SARS-CoV-2 strain (Beta CoV/JS02/Human/2020, GISAID ID: EPI-ISL-411952) used in the neutralization assay was isolated from the throat swab sample of a COVID-19 infected patient in Jiangsu Province, China. The SARS-CoV-2 strain adapted for infecting mouse (BetaCoV/Beijing/IMEBJ05-P6/2020, GISAID ID: GWHACFH01000000) used in the SARS-COV-2 challenge experiments was maintained in the Virology laboratory of Institute of Microbiology and Epidemiology, Academy of Military Medicine. The virus was propagated *via* three passages in Vero-E6 cells and the harvested culture medium was freeze-thawed for three times. After centrifugation at 3000 g for 10 minutes, the clarified supernatant was filtered through a 0.22 µm filter, aliquoted and stored at -80˚C until further use. The virus titer used in neutralization assay was 10-5.0 TCID50/0.1 ml. All the processes involving infectious SARS-Cov-2 were performed in the Biosafety Level 3 (BSL-3) facility.

**Convalescent human donors**

Peripheral blood samples were collected from 5 COVID-19 convalescing patients admitted in Huai’an No 4 People’s Hospital of Jiangsu province, China. Peripheral blood mononuclear cells (PBMCs) were isolated by using Ficoll-Paque Plus (GE Healthcare) density gradient media according to the manufacturer’s protocol. Briefly, blood samples were diluted with the same volume of phosphate buffered saline (PBS) (Gibco). The diluted blood was slowly transferred over Ficoll-Paque in SepMate-50 tube (Stemcell). After horizontal centrifugation at room temperature at 800 g for 20 minutes, PBMCs were collected and transferred into a new centrifuge tube. Following two steps of washing with PBS, total RNA of PBMCs was extracted by using RNeasy Mini Kit (Qiagen, Valencia, CA) following the manufacturer’s instructions.

**Construction of the phage antibody library pool**

The five samples of RNAs extracted from the PBMCs of five COVID-19 convalescing patients were used as templates for cDNA synthesis by Transcriptor High Fidelity cDNA Synthesis kit (Roche, Mannheim, Germany). The resulting five cDNA samples were mixed equally to form a pool of templates. Full length light chain genes and Fab fragments of heavy chain genes were amplified from the cDNA template pool by PCR using the primer pairs from 5VK, 7VL and 8VH gene family as described elsewhere ([*39*](#_ENREF_39)). Light chain genes were cloned into the phagemid vector pComb3H by Xab I and SacI to form the light chain gene pool. The heavy chain genes were sequentially cloned into the light chain gene pool with enzymes Xho I and Spe I following a standard protocol published previously ([*40*](#_ENREF_40)). By two-step cloning, the final anti-SARS-CoV-2 phage antibody library pool was constructed. The phage antibody library pool was aliquoted and frozen at -80℃ until further use.

**Panning phage antibody library**

Four recombinant proteins, S-ECD, S1-RBD, S1-NTD and S2 (Sino biological, 14311-H20B, 40592-V08B, 40591-V49H-B 40590-V08B), were used for panning the phage antibody library pool following the procedure described previously ([*40*](#_ENREF_40)). Briefly, in the first panning of the antibody library, 50 mcg/mL SARS-CoV-2 S-ECD was coated in the immune tubes and incubated at 4 ℃ overnight. At the end of incubation period, the supernatants were discarded and the tubes were washed with TBST for five times. After this, the phage antibody library pool was added and the tubes were incubated at 37℃ for 2 hours, followed by removal of the supernatant and washing with TBST for five times. Finally, the enriched phage antibody library pool was eluted using 0.1M Glycine-HCl (pH 2.2). Using the same procedure, 20 mcg/mL and 10 mcg/mL of SARS-COV-2 S1 NTD-His or S1 RBD-His or S2-His were used respectively for the second and third panning for screening specific binding antibodies pool by phage display. After three rounds of panning, clones from the specific binding antibodies pool (Fab) were divided into specific antibodies against S1-RBD, S1-NTD or S2.

**ELISA for screening the specific binding antibodies**

96-well enzyme-linked immunosorbent assay (ELISA) plates (Corning, 42592) were coated with purified S1-RBD, S1-NTD or S2 and incubated at 4℃ overnight. The plates were blocked with 100 μL of 1% BSA (Sangon, A600332-0100)/PBS (Gibco, 10010-023) treatment at 37°C for 1 hour, and washed five times with PBST. Then the sample wells were incubated with 50 μL single clones from the specific binding antibodies pool and 50 μL 1% BSA/PBS at 37°C for 1 hour, and the four blank control wells were supplemented with 100 μL 1% BSA/PBS at the same time. Following 5 washes with PBST, 100 μL of a 1:3000 dilution of horseradish peroxidase (HRP) conjugated anti-human Fab (Sigma, A0293) was added and incubated at 37°C for 1 hour. After 5 washes with PBST, 100 μL of 3,3',5,5'-tetramethylbenzidine substrate (Thermo Scientific, 34029) was added and incubated at room temperature for 10 min, following which the color change was monitored at 450 nm by adding 50 μL 2M H_2_SO_4_ to stop the reaction. The cut off value was set as the mean of the blank controls +2 standard deviation ((X) ̅+2SD). The OD 450 value > X ̅+2SD was considered as positive well. In this way, single phage antibody against these antigens could be separated.

**Sequencing and genetic analyses of antibodies**

All the single phage antibodies binding to S1-NTD, S1-RBD and S2 protein of SARS-CoV-2 screened using ELISA were sequenced. Phage plasmid samples were sent to Sangon Biotech company (www.sangon.com) for sequencing the antibody genes. Antibody sequences were run through blast and aligned with homologous sequences of the IMGT (www.imgt.org/) database. Phylogenetic tree of variable regions was constructed by MEGA 6.0 software. The full length of variable region amino acids was aligned by using the web servers of ESPrit 3.0 (http://espript.ibcp.fr/ESPript/ESPript/). The sequence logos of Heavy chain, Lambda chain and Kappa chain were generated using the web server of Weblogo (http://weblogo.berkeley.edu/logo.cgi). Based on the diversity of variable genes by primary sequence alignments and the distance in phylogenetic tree as well as OD values of indirect ELISA, the specific candidate antibodies plasmids were optimized.

**Expression and purification of antibodies**

Heavy and light chain genes of optimized antibody plasmid were cloned into PTT5 vector containing the constant regions of human IgG1, respectively. Eukaryotic expression vectors of heavy and light chains were co-transfected into HEK-293F cells by polyetherimide (PEI, Polysciences, 23966-1). In brief, 500 μg of heavy chain expression plasmids and 500μg of light chain expression plasmids were mixed with 3.0 mL PEI (1 mg/mL) and transfected into 1 L HEK-293F cell culture at a density of 1.5×10^6^ cells/mL. After transfection, HEK-293F cells were kept shaking at 37 °C with 8% CO_2_ for antibody expression. After 7 days, the culture supernatant was harvested by centrifuging at 10,000 g for 10 minutes and filtered through 0.22 μm membrane. Protein A column (GE Healthcare, Sweden) was used for antibody purification by NGC Quest 10 Plus system (Bio-Rad). Concentrations were determined by BCA Protein Assay Kits (Thermo Scientific). The purified antibodies were aliquoted and frozen at -80 ℃ until further use.

**Cross-reaction of antibodies**

Representative purified antibodies selected from each cluster according to sequence analysis results were analyzed for their cross-reactivity by ELISA. 96-well ELISA plates were coated with 200 ng/well of RBD, NTD and S2 from SARS-COV-2 or SARS-COV or MERS-COV and incubated at 4℃ overnight. The plates were blocked with 1% BSA/PBS at 37 ℃ for 1 hour. Specific candidate antibodies were serially diluted from 1:2000 to 1:128000 in 1% BSA/PBS, added to the plates and incubated at 37℃ for 45 minutes. After washing with PBST for five times, each well was further incubated with the HRP-anti-human IgG or anti-human Fc secondary antibody (1:3000, Sigma) for 30 minutes at 37 ℃. Subsequently, the plates were washed five times with PBST, and 100 μL of 3,3',5,5'-Tetramethylbenzidine substrate was added into each well. After 15 minutes of incubation at room temperature, the reaction was stopped by adding 50 μL of 2 M H_2_SO_4_ solution and the wells were analyzed by reading their absorbance at 450 nm wavelength. Results were evaluated by calculating the difference between the OD values of the test and control wells.

**Surface plasmon resonance**

SARS-CoV-2 S trimer, SARS-CoV-2 RBD or SARS-CoV-2 NTD was immobilized onto a CM5 sensor chip surface using the NHS/EDC method to a level of ~600 response units (RUs) using Biacore 8K or Biacore T100 (GE Healthcare) and PBS as running buffer (supplemented with 0.05% Tween-20). FC01, FC08, FC11, FC05, FC06, FC07, FC118, FC120, FC122 and FC124, which were purified and diluted, were injected in concentration from high to low. The antibody-binding responses were measured, and these antibodies except FC08 were regenerated with 10 mM Glycine, pH 1.5 (GE Healthcare), while the FC08 was regenerated with 30 mM NaOH. The apparent binding affinity (K_D_) for individual antibody was calculated using Biacore T100 or Biacore 8K Evaluation Software (GE Healthcare). In the same way, SARS-S trimer was immobilized onto a CM5 sensor chip surface and individual antibody -binding response was measured and analyzed using the same software.

For the competitive binding assays, the first sample flew over the chip at a rate of 20 µl/min for 120 s, then the second sample was injected at the same rate for another 120s. All antibodies were evaluated at saturation concentration of 500 nM, except for FC05 (1000 nM), FC118 (1000 nM) and FC124 (1000 nM). All antibodies except FC08 were regenerated with 10 mM Glycine, pH 1.5 (GE Healthcare). FC08 was regenerated with 30 mM NaOH. The response units were recorded at room temperature and analyzed using the same software as mentioned above.

**Authentic SARS-CoV-2 neutralization assay in Vero-E6**

African green monkey kidney Vero E6 cell line was purchased from the Cell Resources Center of Shanghai Institute of Life Science, Chinese Academy of Sciences (Shanghai, China) and cultured in DMEM medium (Gibco Invitrogen, no. 12430-054) containing 10% fetal bovine serum (FBS; Gibco Invitrogen) and incubated at 37 °C with 5% CO2 atmosphere. The viruses were propagated in Vero E6 cells, and viral titers were determined as 50% tissue culture infective dose (TCID50) using microscopic observation of cytopathic effects (CPE). Serum from convalescence patients confirmed with COVID-19 was used as positive controls, and healthy human serum was used as negative control. Serum samples were heat‐inactivated for 30 minutes at 56 ℃, and were then diluted by two‐fold serial dilutions, starting from 1:10. An equal volume of viral solution containing 100 TCID50 of SARS‐CoV‐2 was added to the serum, and the serum‐virus mixture was incubated for 1 hour at 37°C with 5% CO2. After incubation, 100µL of the serum‐virus mixture at each dilution was added to a cell plate containing a semi‐confluent Vero E6 monolayer in duplicate. Cells were incubated at 37°C in a humidified atmosphere containing 5% CO_2_. At day 3~7 post-infection, the plates were inspected daily for CPE by an inverted optical microscope. The highest serum dilution that protected more than the 50% of cells from CPE was taken as the neutralization titre, calculated by using Reed-Muench method.

**Protein expression and purification**

The cloning and production of SARS-CoV-2 RBD (residues 319-541, GenBank: MN908947.3), and SARS-CoV-2 S trimer (residues 1–1208, GenBank:MN908947.3) were performed following the protocol published previously([*41*](#_ENREF_41)). The gene of SARS-CoV-2 NTD (residues 1-305, GenBank: MN908947.3) was synthesized and subcloned into the mammalian expression vector pCAGGS with a C-terminal 2×StrepTag to facilitate protein purification. Briefly, SARS-CoV-2 NTD, SARS-CoV-2 RBD and SARS-CoV-2 S trimer were expressed by transient transfection of HEK Expi 293F cells (Gibco, Thermo Fisher) using polyethylenimine Max Mw 40,000 (PEI MAX 40K, Polysciences). The target protein was purified from clarified cell supernatants 3 days post-transfection using StrepTactin resin (IBA). The resulting protein samples were further purified by size-exclusion chromatography using a Superose 6 10/300 column (GE Healthcare) or a Superdex 200 10/300 Increase column (GE Healthcare) equilibrated with 20mM Tris, 200 mM NaCl, pH 8.0. The purified SARS-CoV-2 NTD and SARS-CoV-2 RBD were used as the immunogens for candidate vaccine evaluation.

**Production of Fab fragment**

The FC01, FC05, FC06, FC07, FC08, FC11, FC118, FC120, FC122, FC124 and D14 Fab fragments were generated using a Pierce FAB preparation Kit (Thermo Scientific). Briefly, the antibody was mixed with immobilized-papain and then digested at 37 ˚C for 3-4 h. The Fab was separated from the Fc fragment and undigested IgGs by protein A affinity column and then concentrated for analysis.

**Negative stain**

The SARS-CoV-2 S trimer and S-Fab (FC01, FC05, FC08, FC11) complexes samples were diluted to an appropriate concentration (~0.02 mg/mL) and dropped onto a glow-discharged carbon-coated grid. After rinsing, the grid was stained with 2% uranyl formate and then loaded onto a 120 kV TEM for examination.

**Cryo-EM sample preparation and data collection**

Purified S trimer protein was mixed with FC05 Fab fragments at 1:2 molar ratio and incubated for 5 min on ice. For RBD-FC08-D14-hACE2 complex formation, the RBD, Fab of FC08, Fab of D14 and hACE2 were mixed at the ratio of 1: 1.2: 1.2: 1. Holy-carbon gold grid (Cflat R1.2/1.3 mesh 300) were freshly glow-discharged with a Solarus 950 plasma cleaner (Gatan) for 30s. A 3 μL aliquot of the mixture complex (0.8mg/ml) was transferred onto the grids, blotted with filter paper at 22℃ and 100% humidity, and plunged into the ethane using a Vitrobot Mark IV (FEI). For S trimer-FC05 complex and RBD-FC08-D14-hACE2 complex, micrographs were collected at 300 kV using a Titan Krios microscope (Thermo Fisher), equipped with a K2 detector (Gatan, Pleasanton, CA), using SerialEM automated data collection software ([*42*](#_ENREF_42)) Movies (32 frames, each 0.2 s, total dose 60 e^−^Å^−2^) were recorded at final pixel size of 1.04 Å with a defocus of between -1.5 and -2.7 μm.

**Image processing**

For SARS-CoV-2 S trimer-FC05-Fab complex, a total of 2,746, micrographs were recorded. Firstly, the raw data were processed by MotionCor2, which were aligned and averaged into motion-corrected summed images. Out of these, the defocus value for each micrograph was determined using Gctf ([*43*](#_ENREF_43)). Micrographs of high quality were selected for further processing. Then particles were picked and extracted for two-dimensional alignment ([*44*](#_ENREF_44)). The SARS-CoV-2 trimer (PDB ID: 6VSB) was used as a reference for three-dimensional classification. After the refinement and post processing, the overall resolution of SARS-CoV-2 S trimer-FC05 was up to 3.4 Å, on the basis of the gold-standard Fourier shell correlation (threshold = 0.143) ([*45*](#_ENREF_45)). To improve the resolution of FC05 Fab binding interface, the entire particle stack were combined and we used the block-based reconstruction strategy ([*46-48*](#_ENREF_46)) for focusing classification and refinement. A local reconstruction focusing on the interface around NTD-RBD-Fab region was carried out. The density map for the binding interface could be improved further by local averaging of the NTD-Fab equivalent copies present in different classes, finally yielding a resolution of 3.9 Å for the interface. The quality of the local resolution was evaluated by ResMap ([*49*](#_ENREF_49)).

For RBD-FC08-D14-hACE2 complex, a total of 3,104, micrographs were recorded. Similar to the previous complex, the raw data were processed by MotionCor2. Then, the defocus value for each micrograph was determined by Gctf, and particles were picked for two-dimensional alignment. Next, the well-defined particles were selected for reconstruction in Relion3.0. After the refinement and post processing, the overall resolution of RBD-FC08-D14-hACE2 complex was up to 3.6 Å, on the basis of the gold-standard Fourier shell correlation (threshold = 0.143).

**Model building and refinement**

The atomic model of FC05 and FC08 Fab was initially predicted by Phyre2 server ([*50*](#_ENREF_50)). The FC05 Fab/H014 (PDB ID: 7CAH) and the *apo* SARS-CoV-2 S trimer (PDB ID: 6VSB) structures were manually docked into the refined maps of SARS-CoV-2 S trimer-FC05 using UCSF Chimera and further corrected manually by real-space refinement in COOT. Likewise, the SARS-CoV-2 RBD-hACE2 (PDB ID: 6M0J) structure was docked into the map of RBD-FC08-D14-hACE2 complex. The atomic models were further refined by positional and B-factor refinement in real space using Phenix. Validation of the final model was performed with Molprobity. The data sets and refinement statistics are reported in table S2.

**Live virus challenge in mice**

Female, 6~8 weeks old BALB/c mice were purchased from Charles River Laboratories, Beijing, China. BALB/c mice were randomly assigned to prophylactic, or therapeutic groups or a PBS control group, with three mice per group. All the mice were anaesthetized with thibromoethanol before intranasal inoculation of a mouse adapted strain of SARS-CoV-2 (BetaCoV/Beijing/ IMEBJ05-P6/2020). To examine the therapeutic efficacy of human mAbs, mice were intranasally infected with 30 μl of 2×10^4^ PFU/ml of the virus two hours before passive immunization with either 20mg/kg of the human mAb FC05, or FC08, or a mixture of FC05 and FC08 *via* an intraperitoneal injection. To examine the prophylactic efficacy against SARS-CoV-2 infection, human mAbs FC05, or FC08, or a mixture of FC05 and FC08 at 20mg/kg were administered into mice 12 hours before and 2 hours after intranasal challenge, while mice in the control group received 100 μl of PBS administered 12 hours before and 2 hours after infection. Mice were euthanized at day 3 post-challenge, and then the lungs as well as tracheas were harvested for analysis. Viral RNA was extracted from the supernatant of lung and trachea tissues by using the QIAamp Viral RNA Mini Kit (Qiagen) and the number of viral RNA copies were measured by using One Step RT-PCR kit (RR064A). The experiments were conducted in adherence with the Chinese National Guidelines for the care of laboratory animals and approved by Ethics Committee for Experimental Animals in Academy of Military Medical Sciences, China. The samples were fixed in 4% paraformaldehyde, dehydrated gradually in ethanol, and then embedded in paraffin and cut into 4µm-thick serial sections. The tissue sections were stained with hematoxylin and eosin (H&E), for the observation of the pathological changes of the tissues under optical microscope.

**Immunization of rabbits with candidate vaccine**

New Zealand rabbits about 3 months old, half male and half female, were randomly allocated into 15 groups, with 4 rabbits in each group. Rabbits received three shots *via* the intramuscular route at day 0, day 14, and day 28, respectively. We immunized these rabbits with the following candidate vaccine formulations: alum- or AS01B-adjuvanted RBD at 5 μg/dose and 20 μg/dose, alum- or AS01B-adjuvanted NTD at 5 μg/dose and 20 μg/dose, alum- or AS01B-adjuvanted RBD+NTD (mixed at 1:1 ratio) at 5 μg/dose (2.5 μg RBD + 2.5 μg NTD), 20 μg/dose (10 μg RBD + 10 μg NTD) and 40 μg/dose (20 μg RBD + 20 μg NTD), respectively, and the sham controls. Each dose of the alum-adjuvanted vaccine contains 500 μg of Al(OH)_3_ as adjuvant. Each dose of the AS01B-adjuvanted vaccine contained 0.5 ml of an AS01-like adjuvant. The serum samples were taken from the rabbits before-, and at day 14, 28, 42 post-immunization, respectively. Serum were inactivated at 56℃ for 30 minutes and diluted in twofold steps in media (DMEM, 10% FBS, DMEM, 1x A/A) in duplicate. We measured the neutralizing antibodies (NAbs) titers in serum using the authentic virus neutralization assay for SARS-CoV-2 as described previously ([*28*](#_ENREF_28)). Cytopathic effect (CPE) of each well was recorded under microscopes, and the neutralizing titer was calculated by the dilution number of 50% protective condition. Weight and skin reactions at the injection site of the rabbits were recorded daily, and the biochemical blood parameters were tested at baseline and day 3, 45 after receiving the first immunization. The experiments were conducted in adherence with the Chinese national guidelines for the care of laboratory animals and approved by the Institutional Animal Care and Use Committee of Jiangsu Provincial Center for Diseases Control and Prevention.

**Animal challenging models**

Medical Biology Research, Chinese Academy of Medical Sciences was responsible for animal procurement, feeding, immunization, peripheral blood collection and challenge. Eight rhesus macaques were randomly divided into two groups, with four animals per group. The animals in the placebo group were intramuscularly injected with 0.5 ml PBS+AS01B; other animals in treatment groups were intramuscularly injected with 0.5 ml vaccine of ReCovR+N (20 μg RBD + 20 μg NTD) and AS01B. Vaccines and placebos were injected intramuscularly into the right thigh of each rhesus monkey. All macaques were immunized at days 0 and 14. A challenge study was conducted 21 days after the second immunization by direct inoculation of SARS-CoV-2 virus of 1×10^5^ TCID_50_ through the intratracheal route under anesthesia. The general symptoms of the animals were observed and recorded every day during the experiment, along with the animal’s body temperature and weight. The animal’s weight was recorded on day 0 before immunization, days 14, 28 after first immunization and every day after the challenge. Peripheral blood samples were collected on day 0 before immunization, days 14, 21, 28, 35 and 43 after first immunization for NAb titer measurements. Blood, nose, throat and anal swabs were collected on day 1-7 after challenge to determine the viral load. The viral loads in the lung, bronchoalveolar lavages (BAL), nasal mucosa, trachea, spleen, pulmonary lymph node and mesenteric lymph node were conducted on day 7 after challenge.


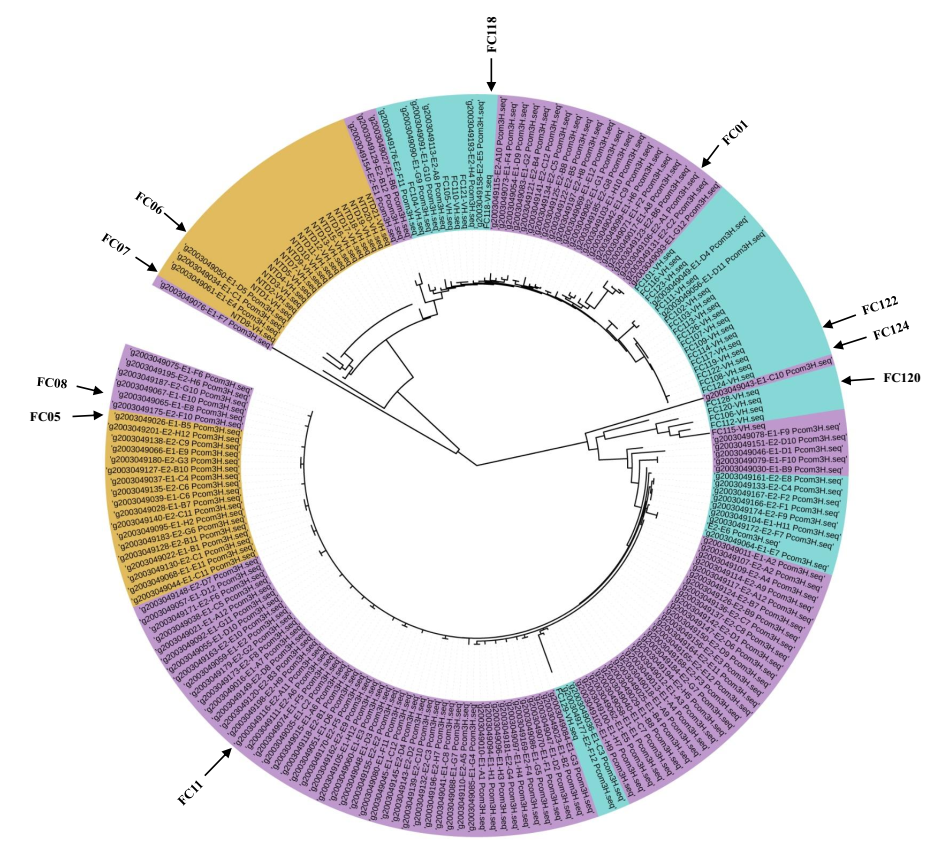


**Fig S1. Maximum-likelihood phylogenetic tree of 202 SARS-CoV-2 mAbs.** Pink, wheat and pale cyan colors represent sequences of mAbs targeting the RBD, NTD and S2 of SARS-CoV-2. 10 representative mAbs are labeled.

**
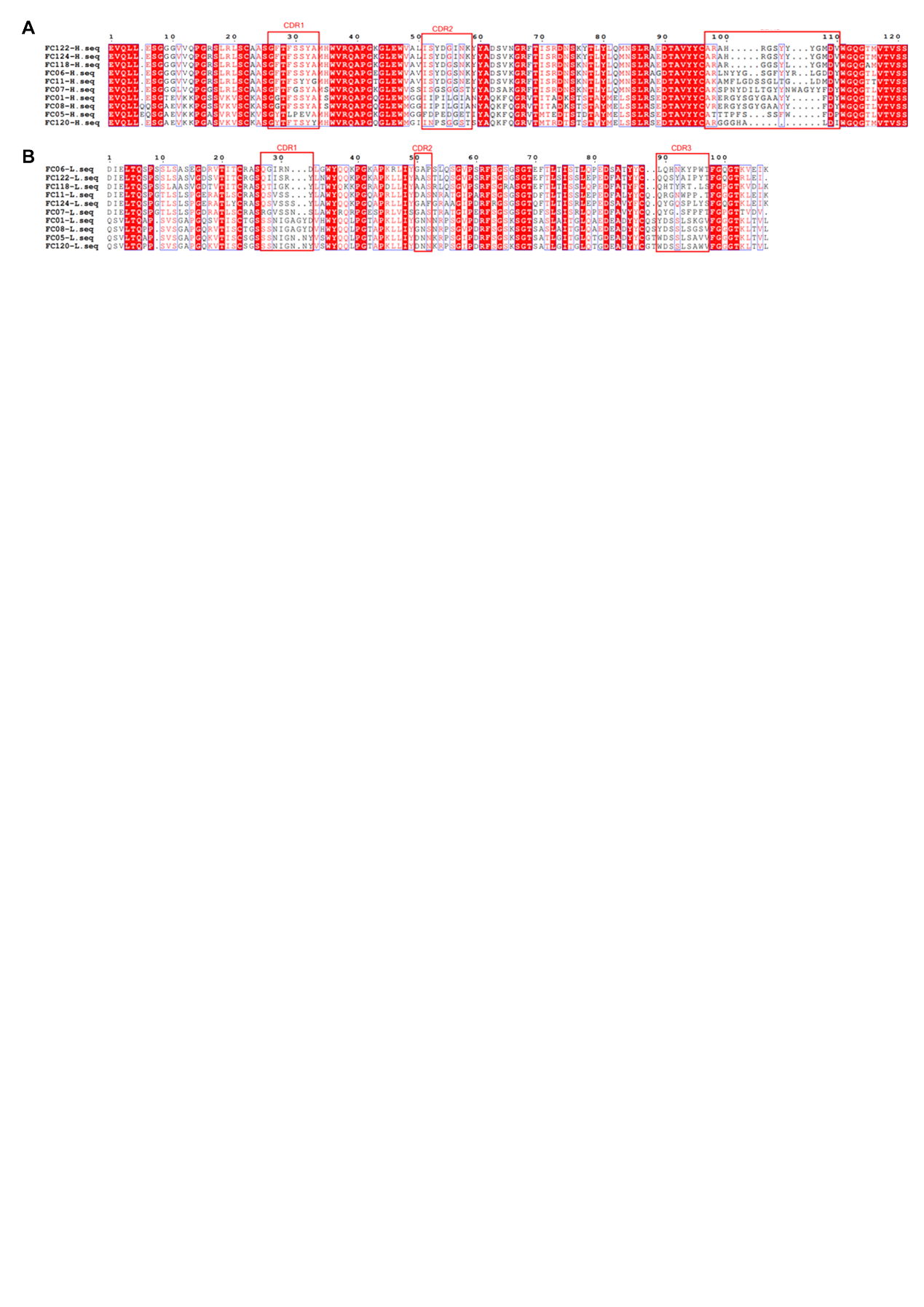
**

**Fig S2. Sequence alignment of 10 representative SARS-CoV-2 mAbs.** Espript ([*51*](#_ENREF_51)) representation of sequence alignment of the variable regions of the heavy chain (A) and light chain (B) from 10 representative SARS-CoV-2 mAbs. CDR loops are marked by red boxes and labeled.

**
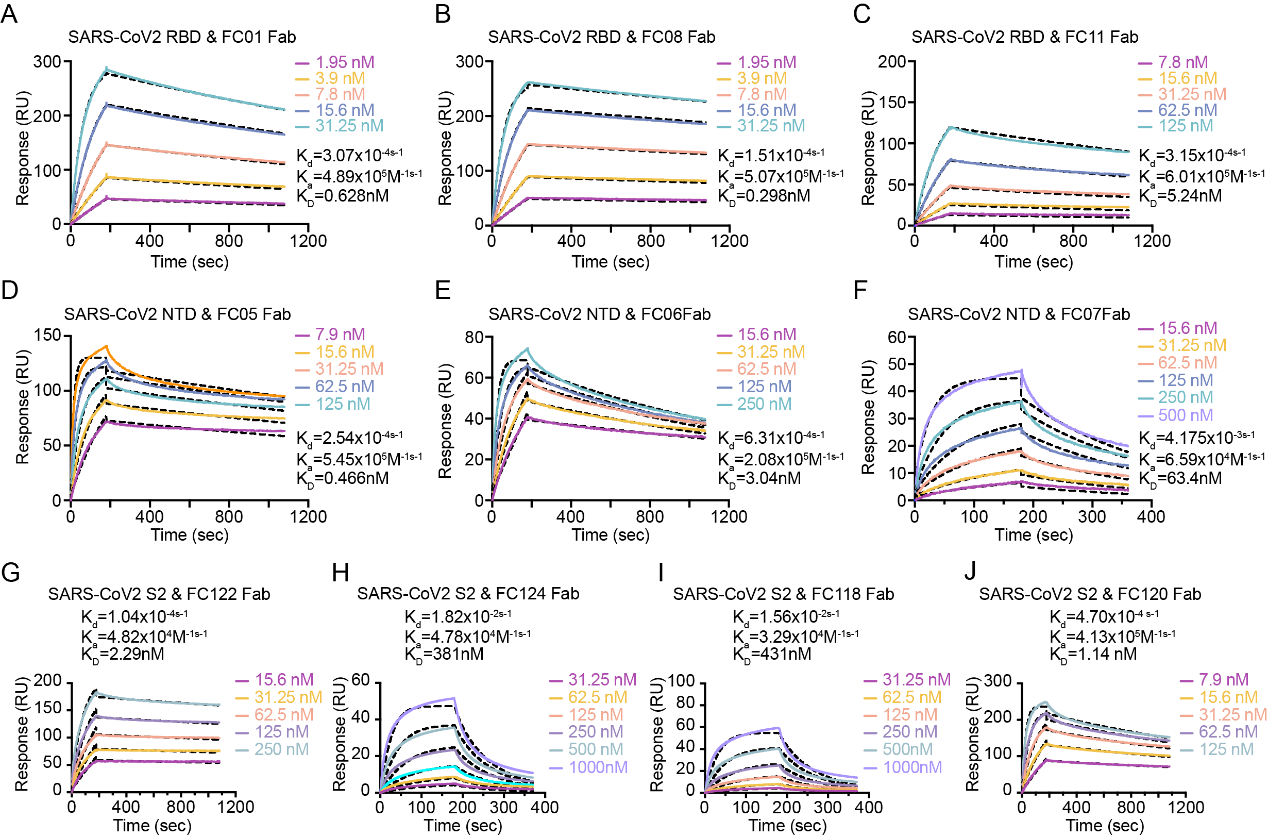
**

**F****ig S3. Binding affinity analysis of 10 representative mAbs to the indicated proteins of SARS-CoV-2 S.** SPR kinetic profiles of the RBD-targeting mAbs, FC01, FC08 and FC11 (A-C); the NTD-targeting mAbs, FC05, FC06 and FC07 (D-F); and S2-directed mAbs, FC122, FC124, FC118 and FC120 (G-J) to SARS-CoV-2 RBD, NTD and S2, respectively.

**
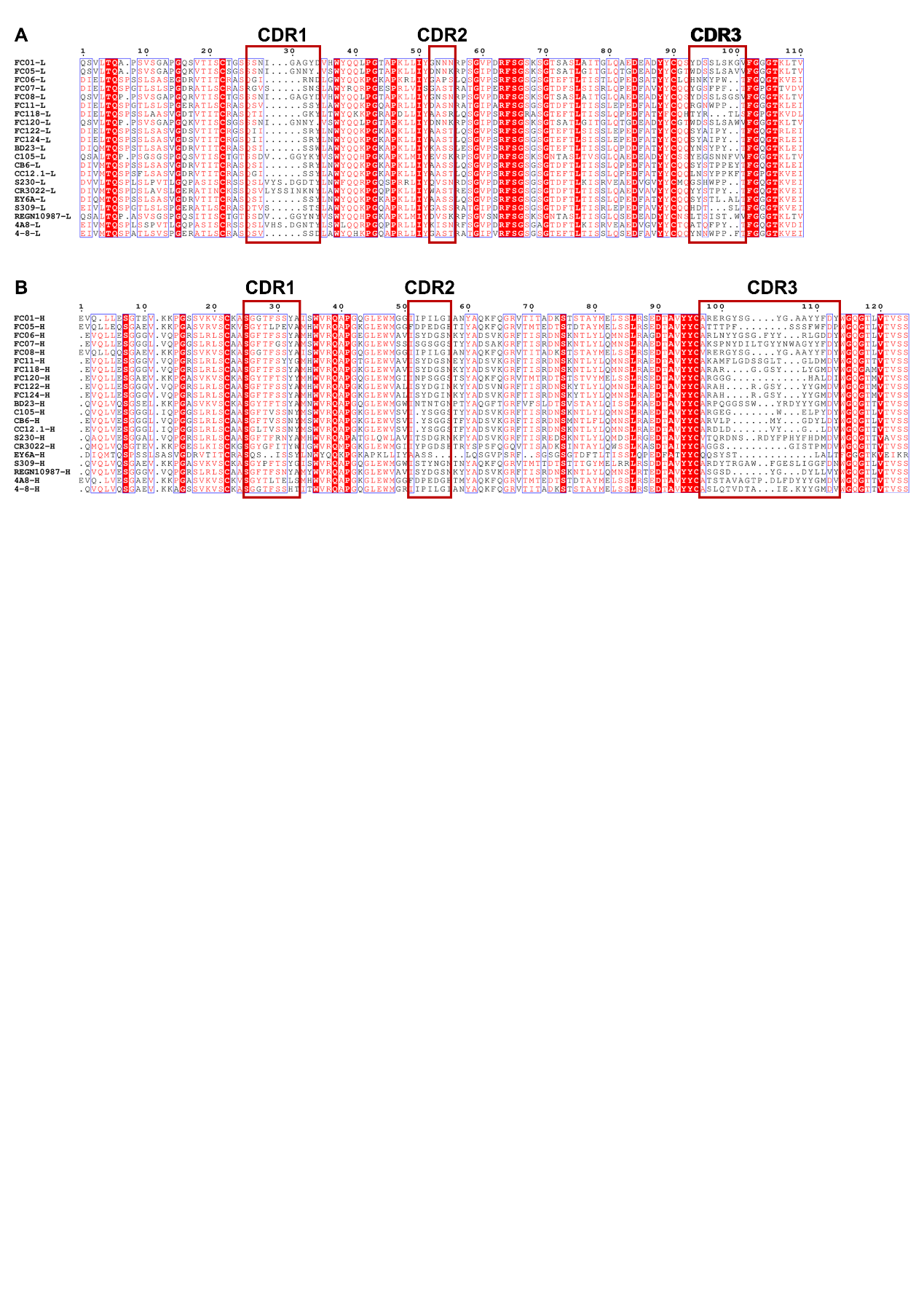
**

**Fig S4. Multiple sequence alignments between our 10 mAbs and typical mAbs recently reported.** Espript ([*51*](#_ENREF_51)) representation of sequence alignment of the variable regions of the light chain (A) and heavy chain (B) from 10 representative SARS-CoV-2 mAbs. CDR loops are marked by red boxes and labeled.

**
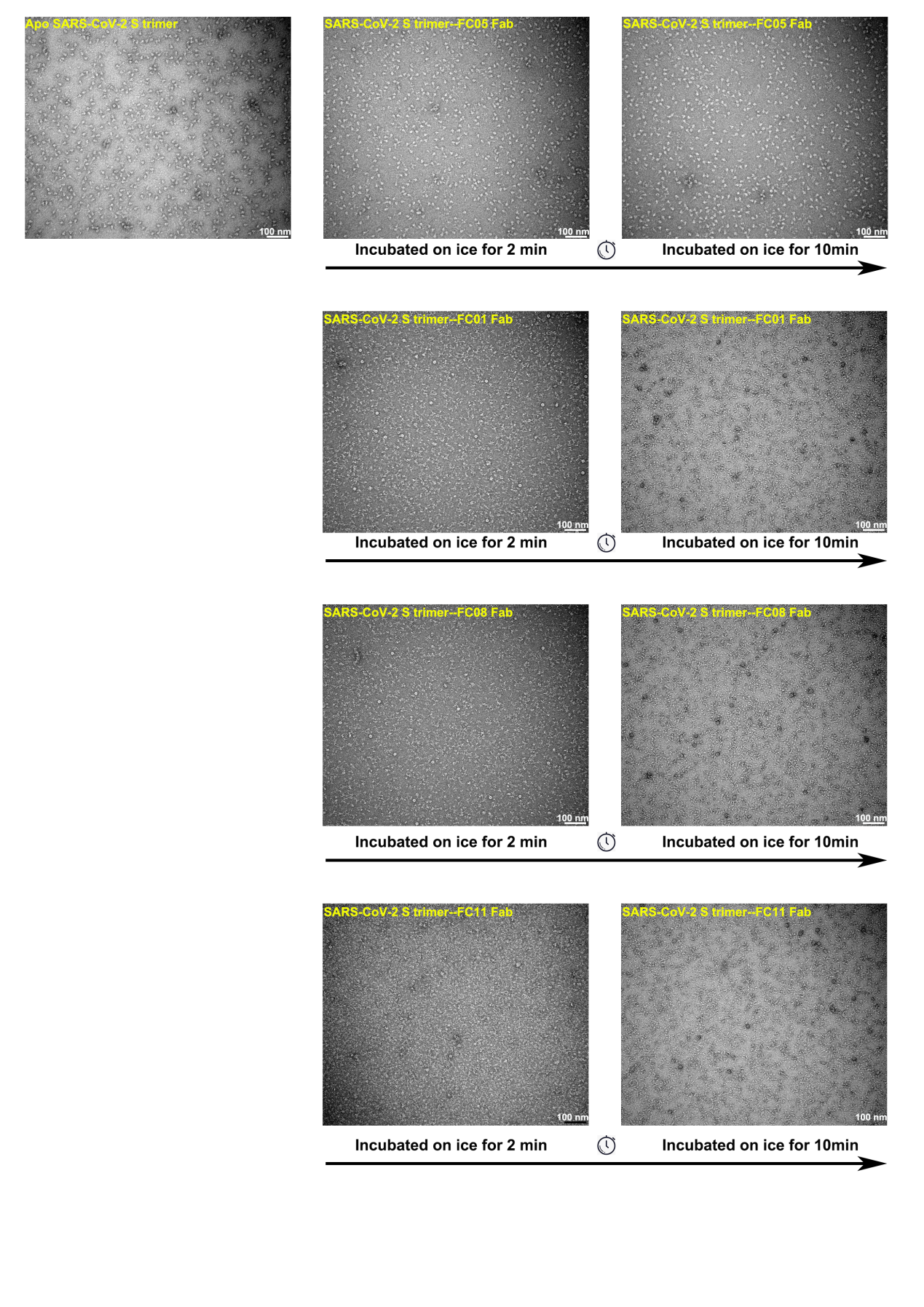
**

**Fig S5. FC01- or FC08- or FC11-mediated destruction of SARS-CoV-2 S trimer visualized by negative staining EM.** The effects of Fabs of 3 RBD-targeting NAbs on the conformational state of SARS-CoV-2 S trimer were analyzed by single-particle EM of negatively stained samples.


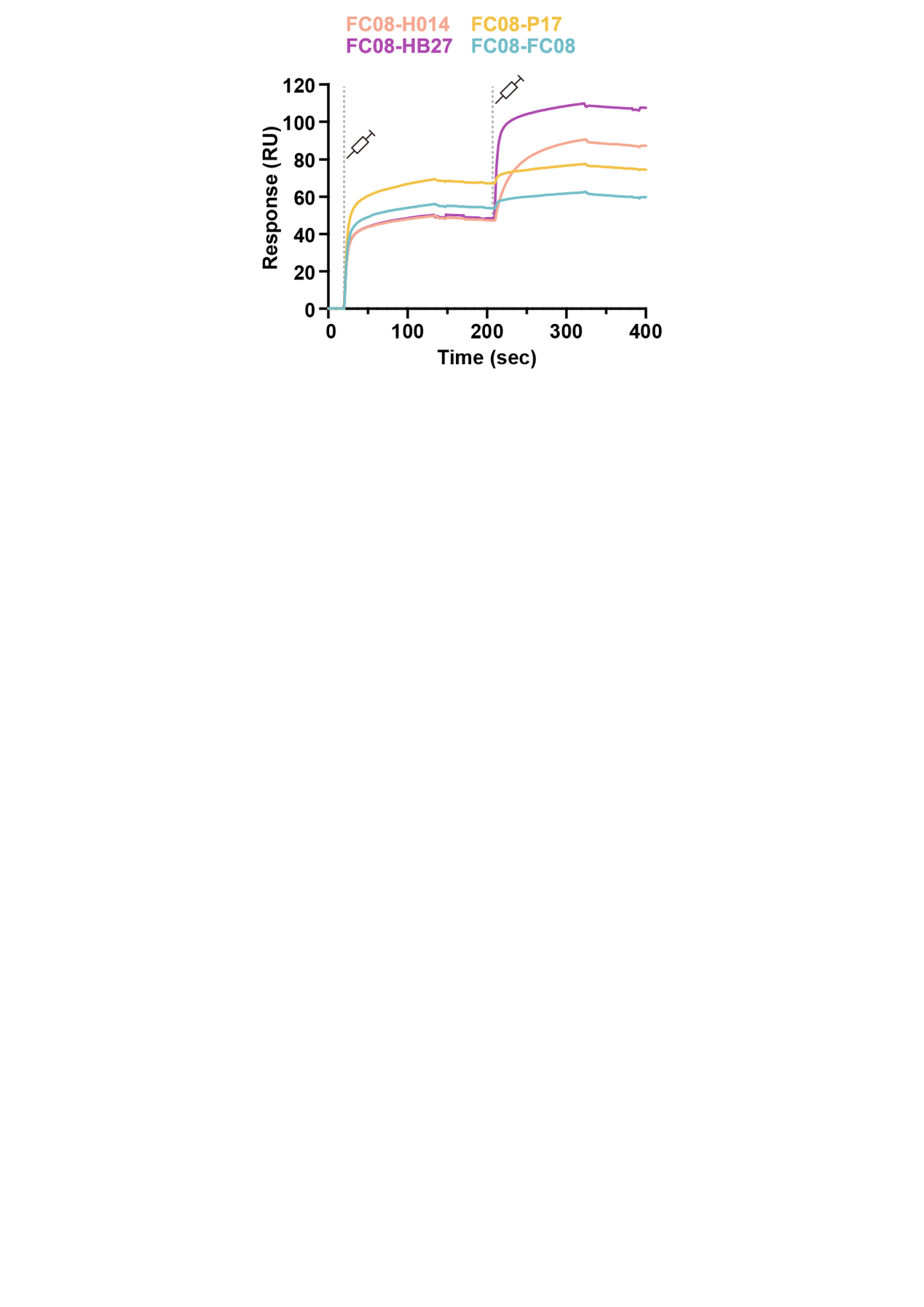


**Fig S6. Competitive SPR-based epitope mapping of FC08 through 3 recently well characterized RBD-targeting SARS-CoV-2 NAbs - H014, HB27 and P17.** The results indicate P17 competes with FC08 for binding to the SARS-CoV-2 S trimer, while H014 and HB27 are capable of simultaneously binding to SARS-CoV-2 S trimer together with FC08.


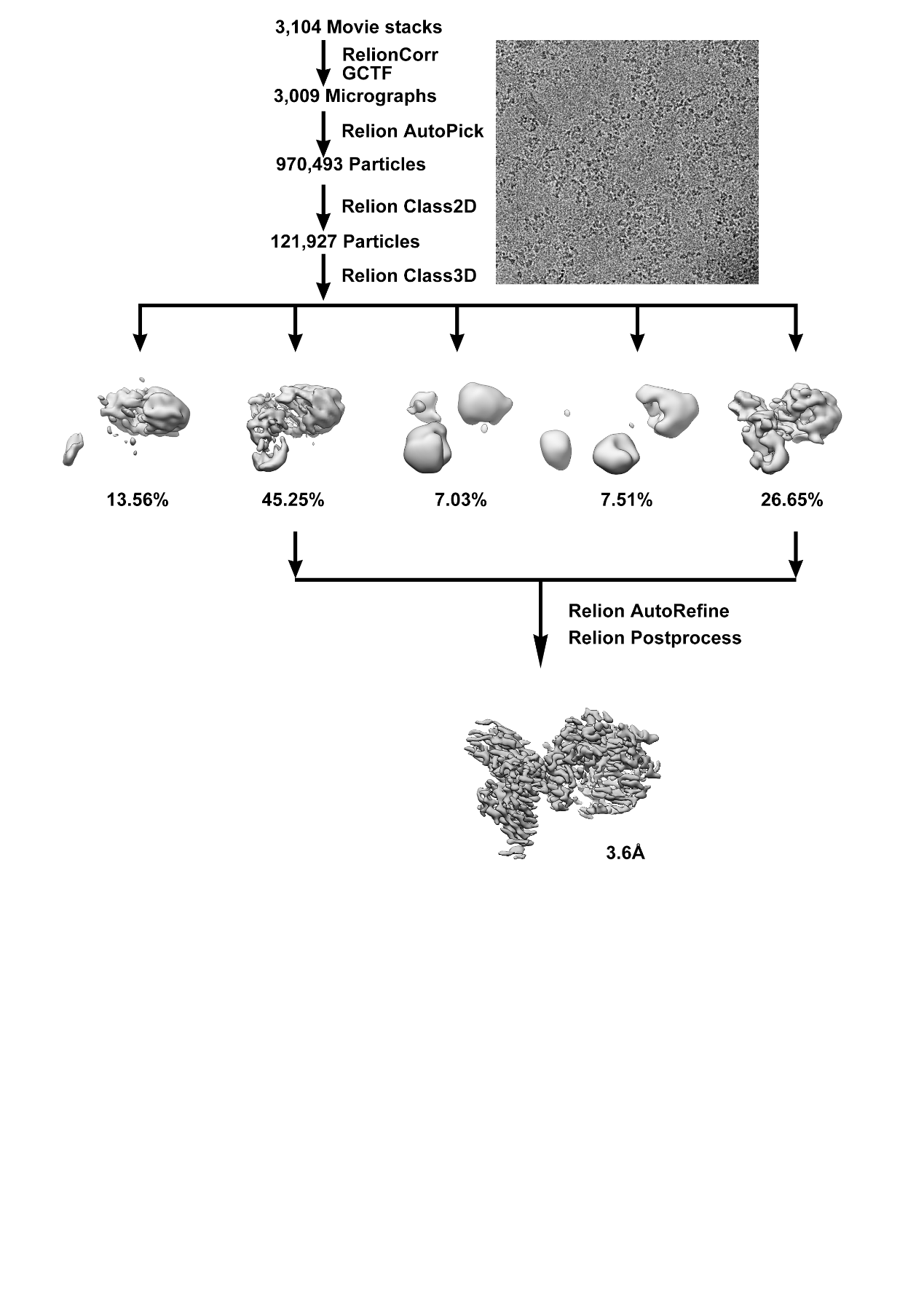


**Fig S7. Flow chart of cryo-EM data processing of SARS-CoV-2 S RBD-FC08-hACE2 complex.**


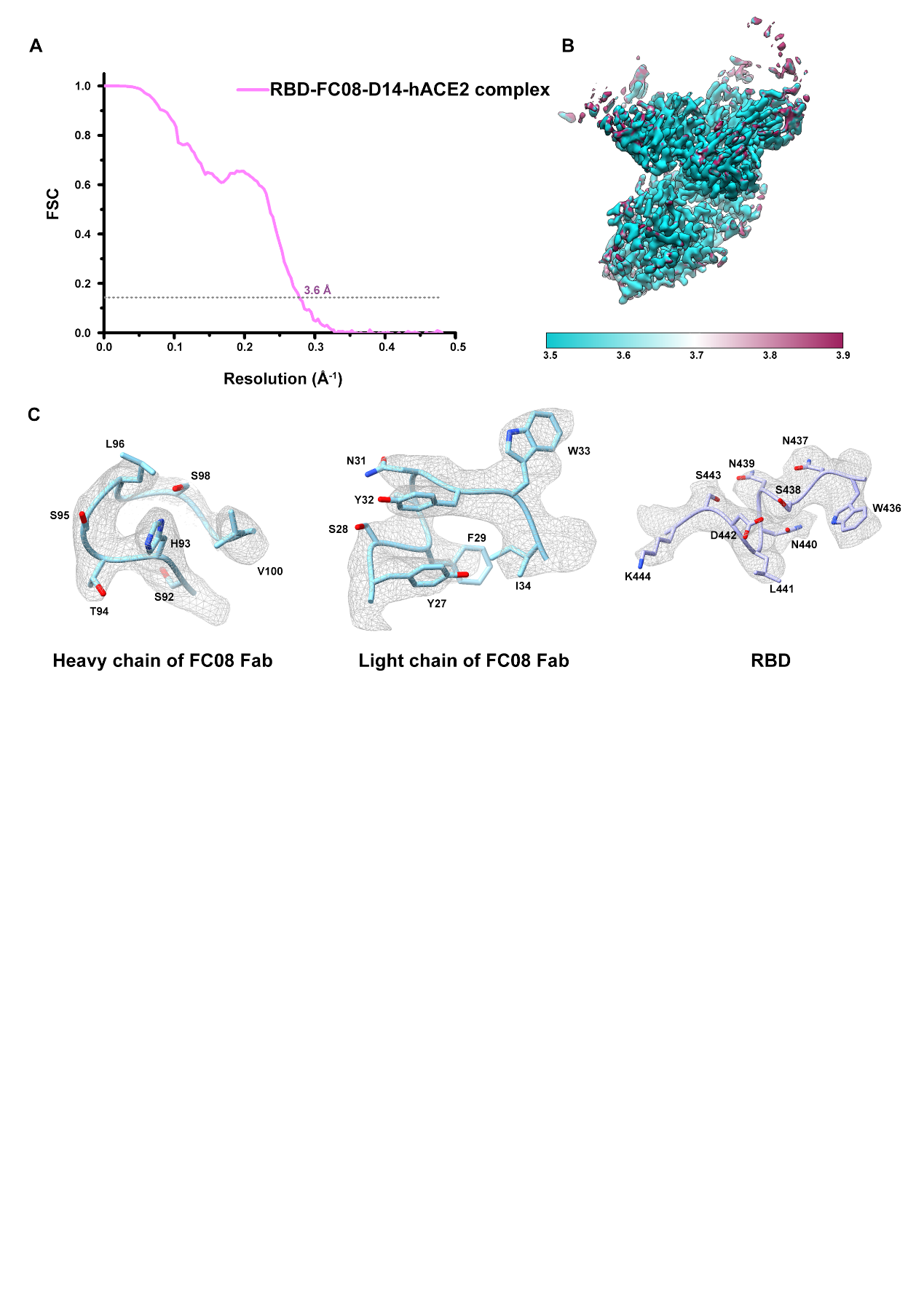


**Fig S8. Resolution evaluation of the EM maps of SARS-CoV-2 S RBD-FC08-hACE2 complex.** (**A**) The gold-standard FSC curves of the final maps. (**B**) Local resolution assessments of cryo-EM maps. (C) Cryo-EM maps of RBD-FC08-D14-hACE2complex. The enlarged panels show the density maps (mesh) and related atomic models. Residues are shown as sticks, oxygen, nitrogen and sulfurs atoms are colored in red, blue and yellow, respectively.


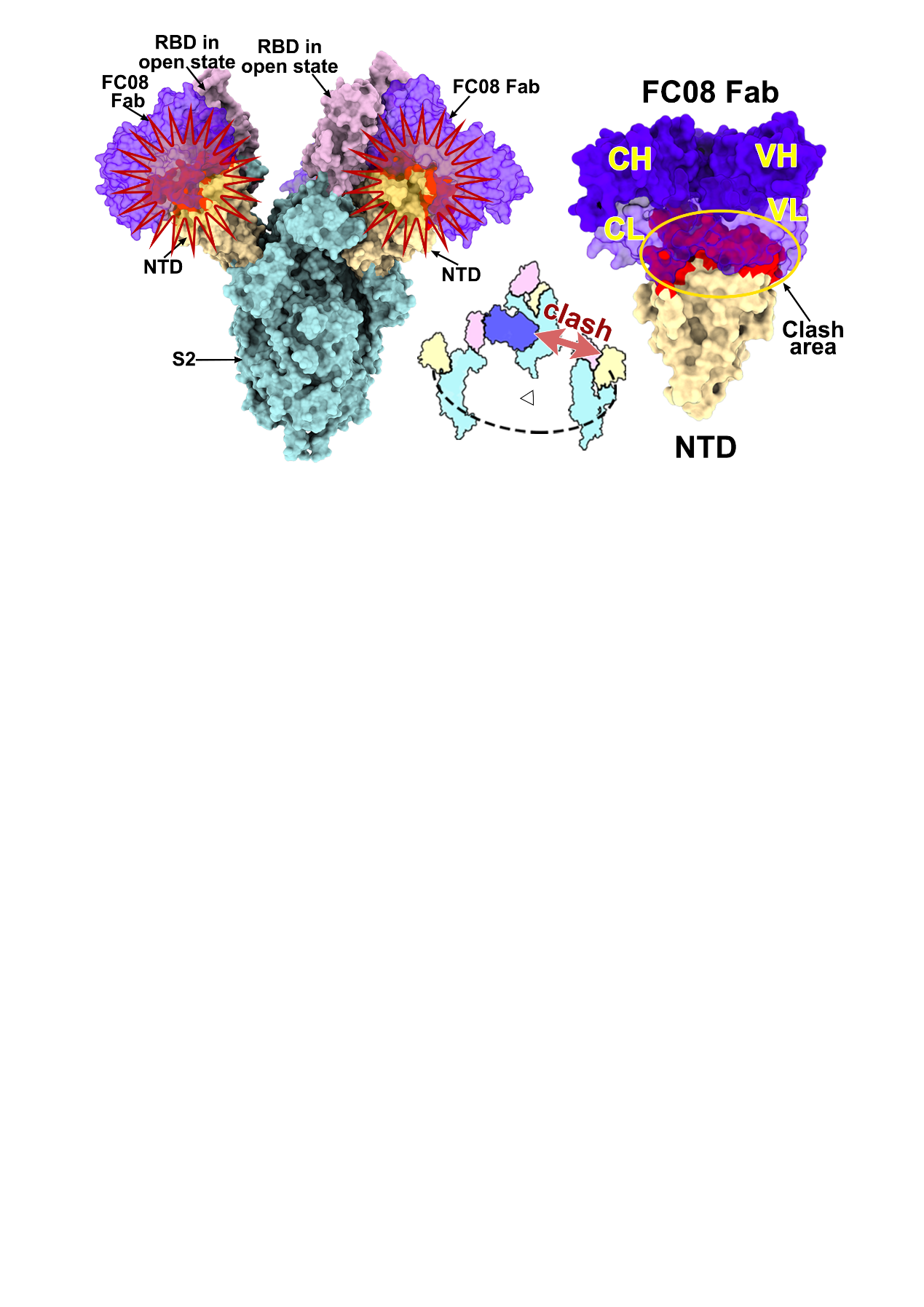


**Fig S9. Superposition model of FC08 binding to SARS-CoV-2 S trimer.** Domains of S2 and NTD are colored in cyan and yellow, respectively. The clash area on NTD was colored by red.


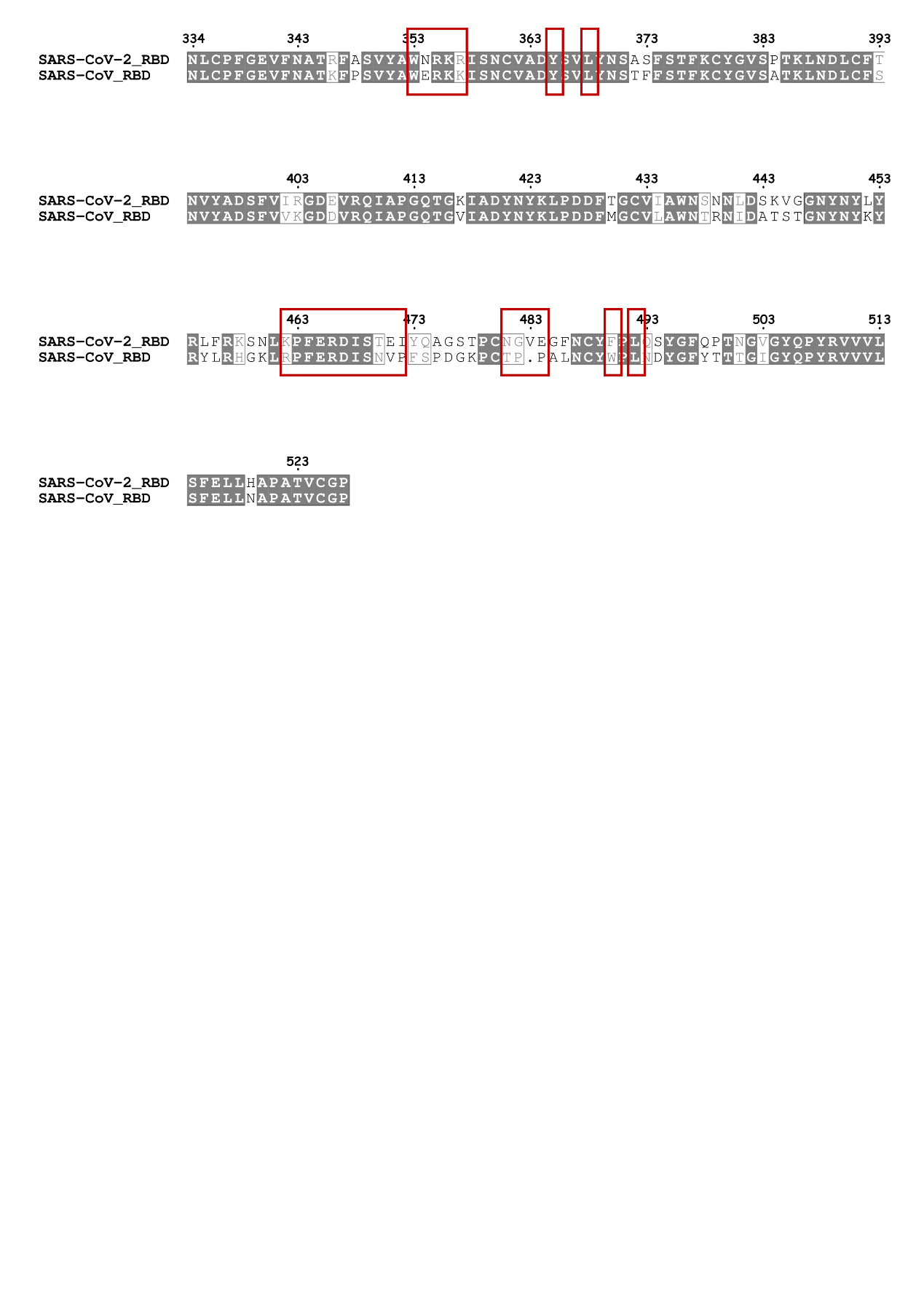


**Fig S10. Sequence alignment of the RBD of SARS-CoV and SARS-CoV-2.** Clustal W was used for sequence alignments, the key residues in the SARS-CoV-2 RBD involved in the interaction with FC08 Fab were marked by red boxes.


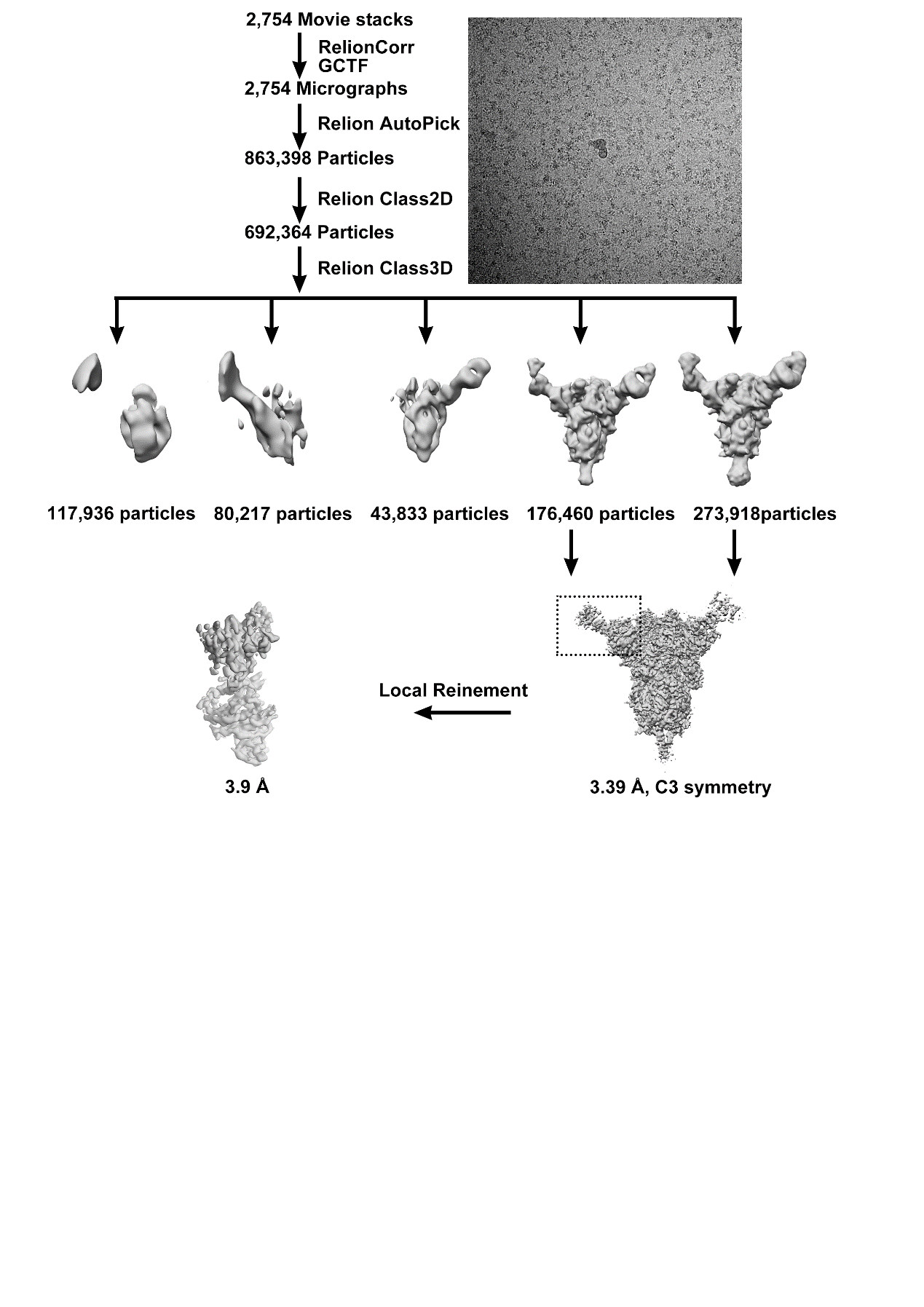


**Fig S11. Flow chart of cryo-EM data processing of SARS-CoV-2 S trimer-FC05 Fab complex.**

**
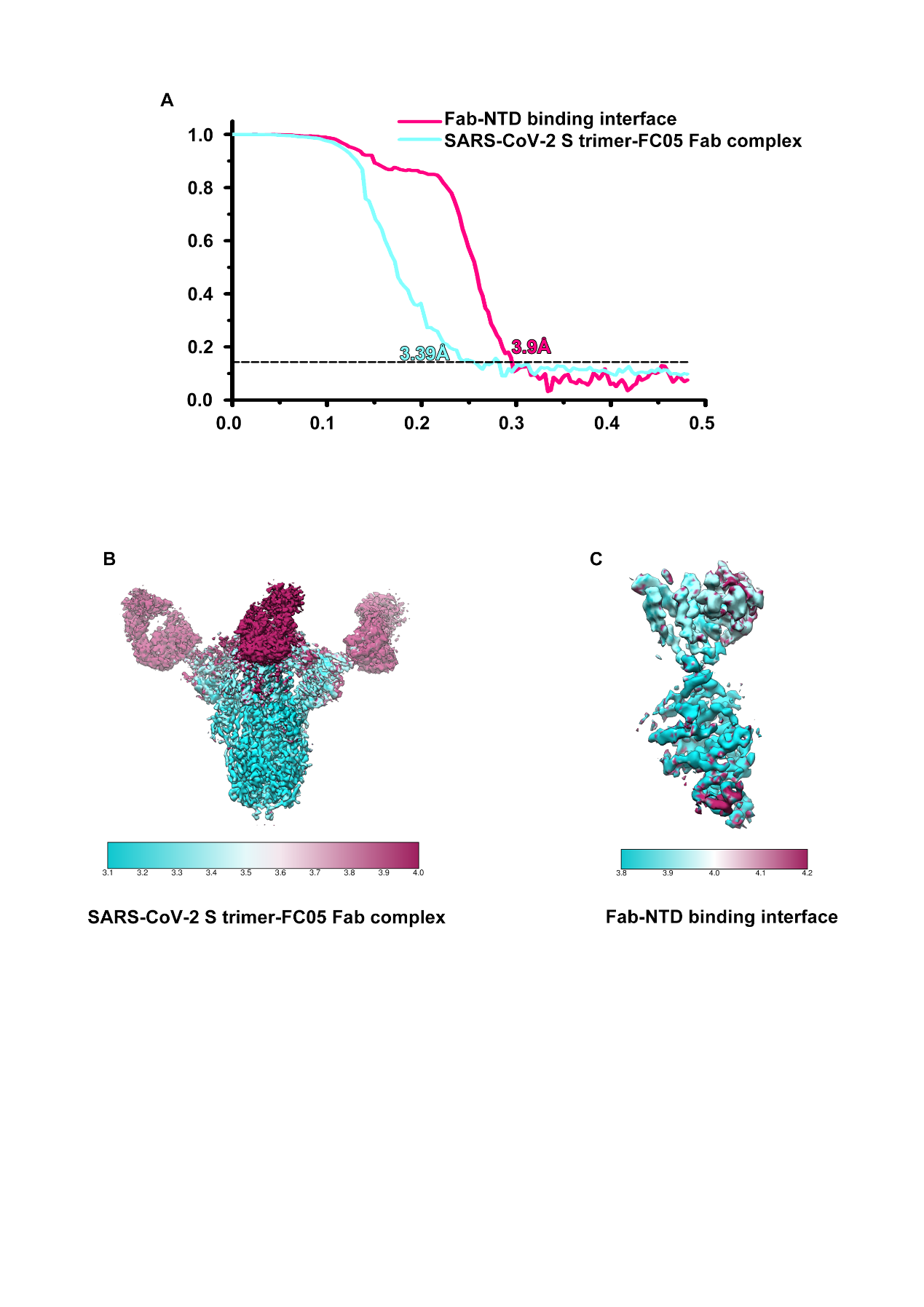
**

**Fig S12. Resolution evaluation of the EM maps of SARS-CoV-2 S trimer-FC05 complexes.** (**A**) The gold-standard FSC curves of the final maps. (**B**) (**C**) Local resolution assessments of cryo-EM maps. Local-resolution evaluation of the maps of the SARS-CoV-2 S trimer in complex with three FC05 Fabs and binding interface using ResMap ([*49*](#_ENREF_49)) are shown.


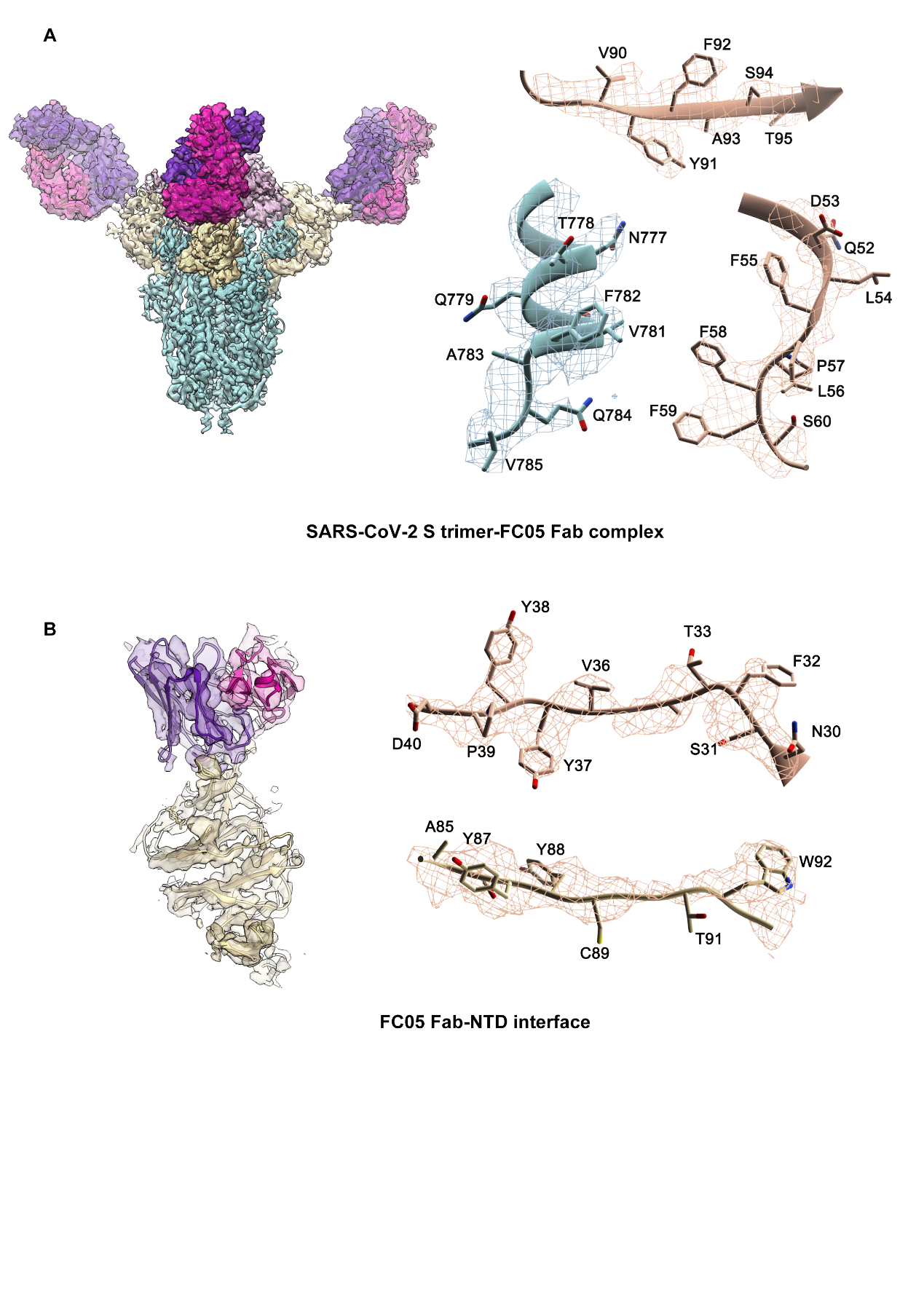


**Fig S13. Density maps and related atomic models.**

Cryo-EM maps of SARS-CoV-2 S trimer in complex with FC05 Fab. The enlarged panels show the density maps (mesh) and related atomic models. Residues are shown as sticks, oxygen, nitrogen and sulfurs atoms are colored in red, blue and yellow, respectively.


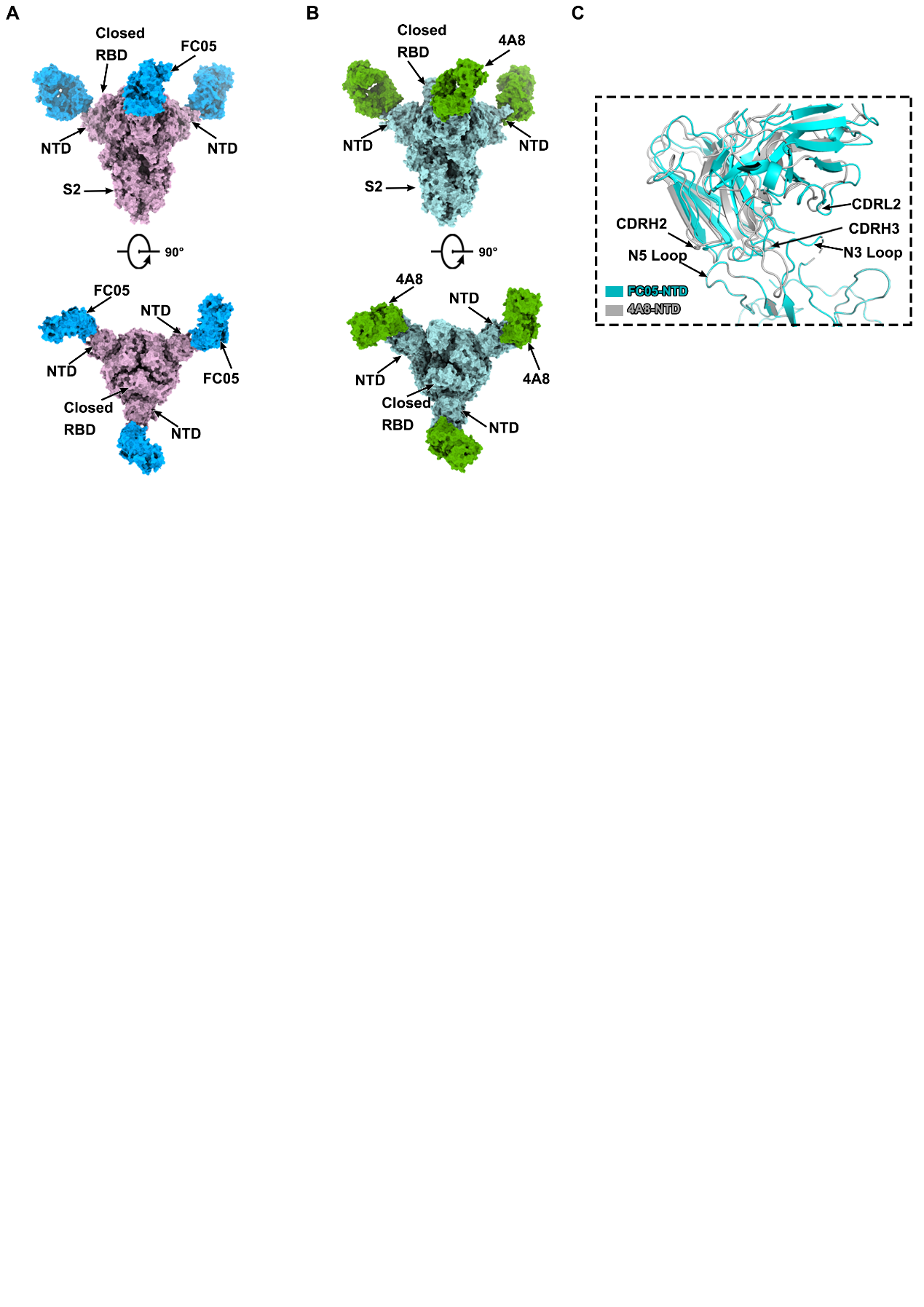


**Fig S14. Comparison of the structures of S-FC05 and S-4A8.** (A) Structure of S-FC05 complex. The S trimer and FC05 Fab were colored in purple and blue, respectively. (B) Structure of S-4A8 complex. The S trimer was colored in cyan. The 4A8 Fab was colored in green. (C) Superimposition of S-FC05 and S-4A8 complex. The S-FC05 complex and S-4A8 complex were colored by cyan and grey, respectively.


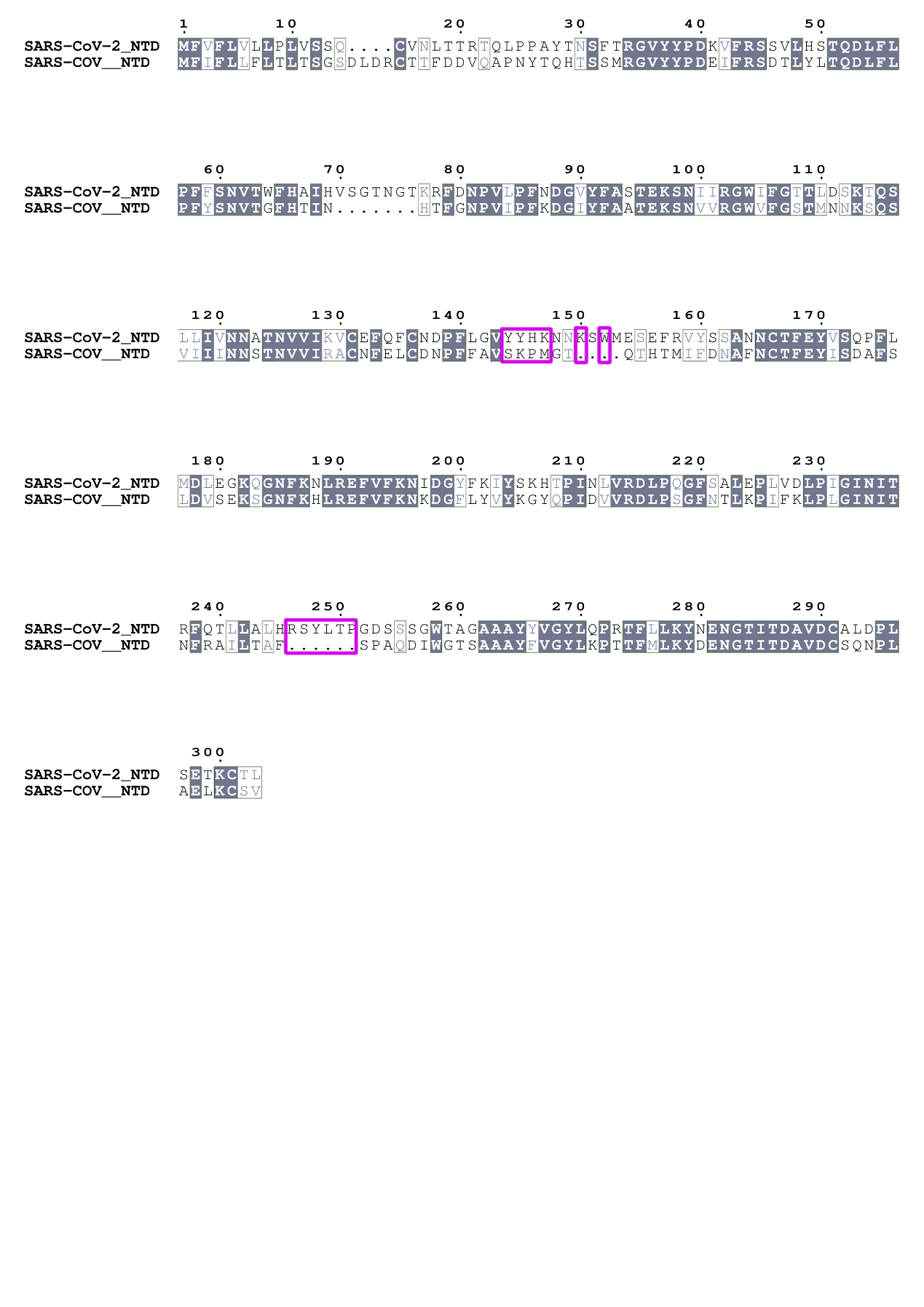


**Fig S15. Sequence alignment of the NTD of SARS-CoV and SARS-CoV-2.** Clustal W was used for sequence alignments, the key residues in the SARS-CoV-2 NTD involved in the interaction with FC05 Fab were marked by purple boxes.


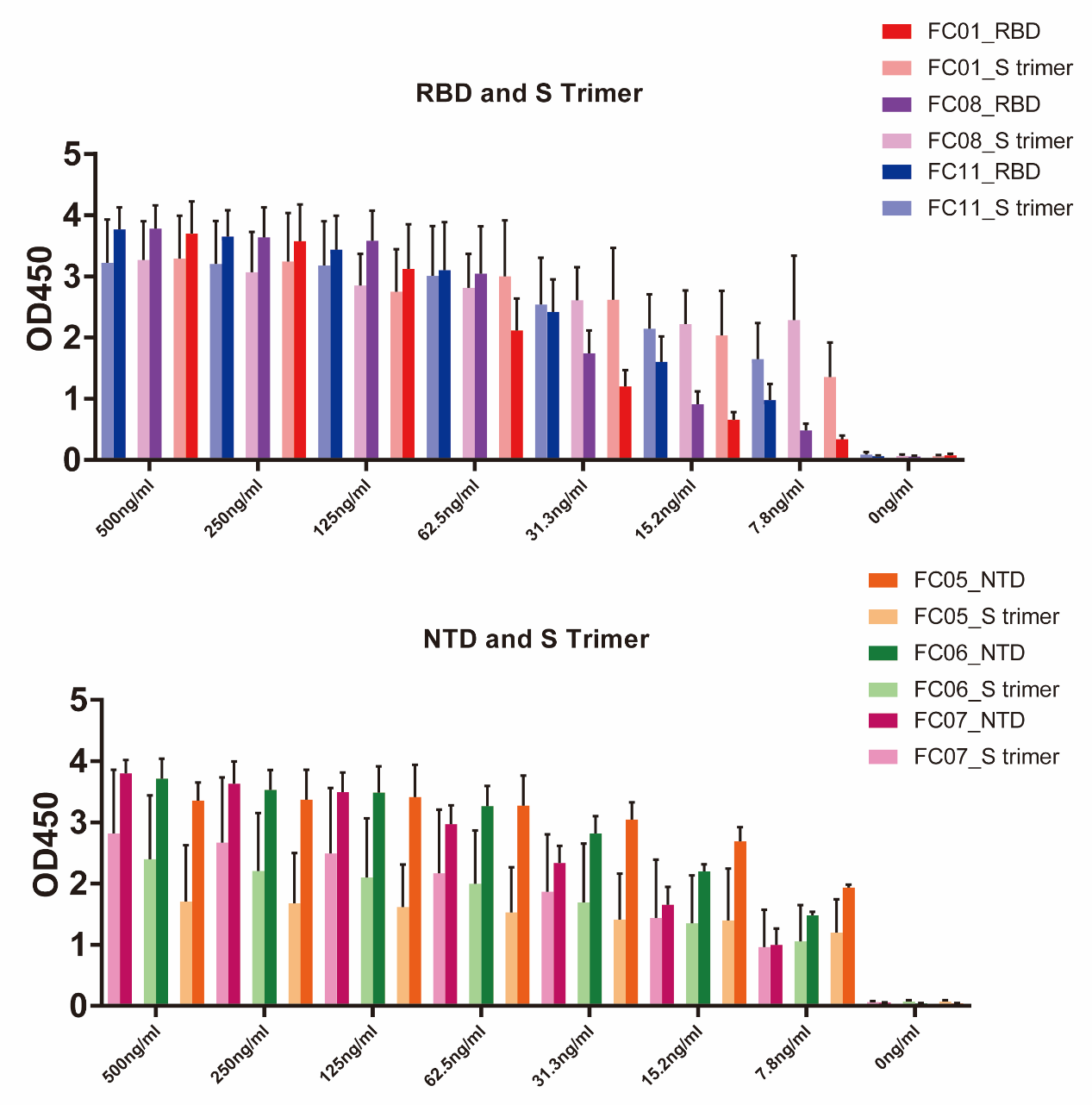


**Fig S16. Activity verification of the recombinant NTD, RBD, or ectodomain of S trimer by ELISA assays using specific NAbs.**


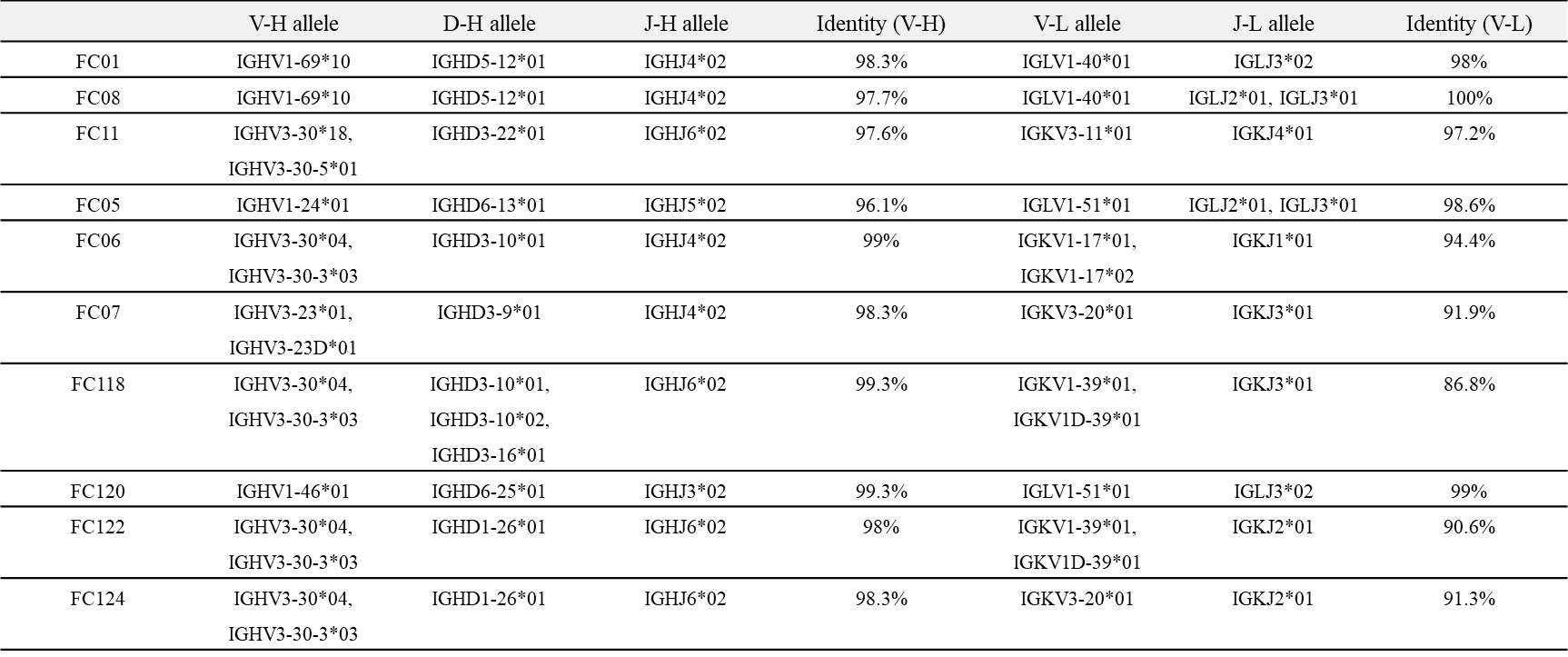
**Table S1 Germline analysis of 10 representative SARS-CoV-2 specific mAbs**

**Table S2 Cryo-EM data collection and atomic model reﬁnement statistics**

| **Data collection and reconstruction statistics** | | | | | |
| --- | --- | --- | --- | --- | --- |
| Protein | SARS-CoV-2 S trimer-FC05 | | | RBD-FC08-D14-hACE2 complex | |
| Voltage (kV) | 300 | | | 300 | |
| Detector | K2 | | | K2 | |
| Pixel size (Å) | 1.04 | | | 1.04 | |
| Electron dose (e^-^/Å^2^) | 60 | | | 60 | |
| Defocus range (μm) | 1.25-2.7 | | | 1.25-2.7 | |
| Final particles | 450,378 | | | 87,543 | |
| Final resolution (Å) | 3.9 | | | 3.6 | |
| **Models refinement and validation statistics** | | | | | |
| Ramachandran statistics | | | | | |
| Favored (%) |  | 91.38 |  |  | 93.56 |
| Allowed (%) |  | 8.41 |  |  | 6.28 |
| Outliers (%) |  | 0.21 |  |  | 0.16 |
| Rotamer outliers (%) |  | 0.18 |  |  | 0.28 |
| R.m.s.d | | | | | |
| Bond lengths (Å) |  | 0.02 |  |  | 0.02 |
| Bond angles (°) |  | 1.26 |  |  | 1.18 |

**T****able S3. Residues of FC08 Fab fragment interacting with the SARS-CoV-2 RBD at the binding interface (d < 4 Å)**

|  | **S-RBD** | **FC08 Fab** | | | | |  |
| --- | --- | --- | --- | --- | --- | --- | --- |
|  | **Residues** | |  | **Light chain** |  | **Heavy chain** | |
| **β1** | W353 | |  | Y33 |  |  | |
|  | N354 | |  | Y33 |  |  | |
|  |  | |  | Y93 |  |  | |
|  |  | |  | S95 |  |  | |
|  | R355 | |  | A31 | |  | |
|  |  | |  | Y33 |  |  | |
|  |  | |  | S95 |  |  | |
|  | K356 | |  | S95 |  |  | |
|  | R357 | |  | S26 |  |  | |
|  |  | |  | G30 |  |  | |
|  |  | |  | A31 |  |  | |
| **β5** | Y449 | |  |  |  | I58 | |
|  | L452 | |  |  |  | I56 | |
| **Loop**  **Between**  **β5 and**  **β6** | K462 | |  |  |  | Y106 | |
|  | P463 | |  |  |  | Y106 | |
|  | F464 | |  |  |  | Y106 | |
|  | E465 | |  |  |  | G105 | |
|  | R466 | |  | Y33 |  | S104 | |
|  |  | |  |  |  | G105 | |
|  |  | |  |  |  | G107 | |
|  | D467 | |  |  |  | S104 | |
|  | I468 | |  |  |  | G102 | |
|  |  | |  |  |  | G107 | |
|  |  | |  |  |  | A108 | |
|  | S469 | |  |  |  | G102 | |
|  | T470 | |  |  |  | S32 | |
|  |  | |  |  |  | R102 | |
|  | E471 | |  |  |  | Y33 | |
|  | I472 | |  |  |  | S32 | |
|  | N481 | |  |  |  | T29 | |
|  | V482 | |  |  |  | T29 | |
|  |  | |  |  |  | S32 | |
|  | G483 | |  |  |  | T29 | |
|  | V484 | |  |  |  | I35 | |
| **β6** | F490 | |  |  |  | S32 | |
|  |  | |  |  |  | I55 | |
|  | L492 | |  |  |  | I56 | |

**Table S4. Residues of FC05 Fab fragment interacting with the SARS-CoV-2 S trimer at the binding interface (d < 4 Å)**

| **S-NTD** | **FC05 Fab** | | | | |  |
| --- | --- | --- | --- | --- | --- | --- |
| **Residues** | |  | **Light chain** |  | **Heavy chain** | |
| Y144 | |  |  |  | Q32 | |
| Y145 | |  |  |  | P31 | |
|  | |  |  |  | Q32 | |
|  | |  |  |  | P102 | |
|  | |  |  | | F103 | |
| H146 | |  |  |  | P31 | |
| K147 | |  |  |  | L30 | |
|  | |  |  |  | P31 | |
|  | |  |  |  | Q73 | |
| K150 | |  |  |  | G57 | |
| W152 | |  |  |  | F103 | |
| R246 | |  |  |  | G27 | |
|  | |  |  |  | Y28 | |
| S247 | |  |  |  | Y28 | |
| Y248 | |  |  |  | Y28 | |
|  | |  |  |  | Q32 | |
|  | |  |  |  | P102 | |
| L249 | |  |  |  | S106 | |
|  | |  |  |  | W108 | |
|  | |  |  |  | D110 | |
| P251 | |  | S57 |  |  | |

**Supplementary References and Notes**

39. C. F. Barbas, 3rd, A. S. Kang et al., Assembly of combinatorial antibody libraries on phage surfaces: the gene III site. Proceedings of the National Academy of Sciences of the United States of America 88, 7978-7982 (1991); published online EpubSep 15 (10.1073/pnas.88.18.7978).

40. Z. Chen, X. Ren et al., An elaborate landscape of the human antibody repertoire against enterovirus 71 infection is revealed by phage display screening and deep sequencing. mAbs 9, 342-349 (2017); published online EpubFeb/Mar (10.1080/19420862.2016.1267086).

41. Y. W. Yao Sun, Rui Feng, Nan Wang, Dandan Zhu, Lei Wang, Xiaorui Xing, Peng Yang, Yanjun Zhang, Weimin Li and Xiangxi Wang, Structure-based development of three- and four-antibody cocktails against SARS-CoV-2 via multiple mechanisms. Cell Res, (2021).

42. D. N. Mastronarde, Automated electron microscope tomography using robust prediction of specimen movements. Journal of structural biology 152, 36-51 (2005); published online EpubOct (10.1016/j.jsb.2005.07.007).

43. K. Zhang, Gctf: Real-time CTF determination and correction. J Struct Biol 193, 1-12 (2016); published online EpubJan (10.1016/j.jsb.2015.11.003).

44. S. H. Scheres, Processing of Structurally Heterogeneous Cryo-EM Data in RELION. Methods enzymol 579, 125-157 (2016)10.1016/bs.mie.2016.04.012).

45. S. H. Scheres, S. Chen, Prevention of overfitting in cryo-EM structure determination. Nature methods 9, 853-854 (2012).

46. Y. Yang, P. Yang et al., Architecture of the herpesvirus genome-packaging complex and implications for DNA translocation. Protein & cell 11, 339-351 (2020); published online EpubMay (10.1007/s13238-020-00710-0).

47. N. Wang, W. Chen et al., Structures of the portal vertex reveal essential protein-protein interactions for Herpesvirus assembly and maturation. Protein & cell 11, 366-373 (2020); published online EpubMay (10.1007/s13238-020-00711-z).

48. L. P. Dong H, Bai M, Wang K, Feng R, Zhu D, Sun Y, Mu S, Li H, Michiel H, Sun, S, Wang X, Guo H, Structural and molecular basis for foot-and-mouth disease virus neutralization by two potent protective antibodies. Protein & Cell, (2021).

49. A. Kucukelbir, F. J. Sigworth et al., Quantifying the local resolution of cryo-EM density maps. Nature methods 11, 63-65 (2014).

50. L. A. Kelley, S. Mezulis et al., The Phyre2 web portal for protein modeling, prediction and analysis. Nature Protocols 10, 845-858 (2015); published online EpubJun (10.1038/nprot.2015.053).

51. P. Gouet, E. Courcelle et al., ESPript: analysis of multiple sequence alignments in PostScript. Bioinformatics 15, 305-308 (1999).
